# Whitening fruit by CRISPR/Cas9-mediated homoeolog-specific gene editing of *MYB10-1B* in strawberry (*F*. × *ananassa*)

**DOI:** 10.1093/hr/uhaf272

**Published:** 2025-10-15

**Authors:** Man Bo Lee, Yoon Jeong Jang, Hyeondae Han, Kanika Saxena, Youngjae Oh, Jae Yoon Kim, Seonghee Lee

**Affiliations:** Department of Plant Resources, College of Industrial Science, Kongju National University, Yesan 32439, South Korea; Gulf Coast Research and Education Center, Institute of Food and Agricultural Science, University of Florida, Wimcauma, FL 33598, USA; Gulf Coast Research and Education Center, Institute of Food and Agricultural Science, University of Florida, Wimcauma, FL 33598, USA; Vegetable Research Division, National Institute of Horticultural and Herbal Science, Rural Development Administration, Wanju 55365, South Korea; Gulf Coast Research and Education Center, Institute of Food and Agricultural Science, University of Florida, Wimcauma, FL 33598, USA; Vegetable Research Division, National Institute of Horticultural and Herbal Science, Rural Development Administration, Wanju 55365, South Korea; Gulf Coast Research and Education Center, Institute of Food and Agricultural Science, University of Florida, Wimcauma, FL 33598, USA; Fall Creek Farm and Nursery, Inc., 39318 Jasper-Lowell Road, Lowell, OR 97452, USA; Gulf Coast Research and Education Center, Institute of Food and Agricultural Science, University of Florida, Wimcauma, FL 33598, USA; Department of Horticultural Science, Chungbuk National University, Cheongju 28644, South Korea; Department of Plant Resources, College of Industrial Science, Kongju National University, Yesan 32439, South Korea; Gulf Coast Research and Education Center, Institute of Food and Agricultural Science, University of Florida, Wimcauma, FL 33598, USA

## Abstract

Fruit color is a key quality trait in strawberry breeding and cultivar development, as it directly influences consumer preference and marketability. Anthocyanins are the pigments responsible for the red coloration in strawberries, and the transcription factor *MYB10* gene plays a crucial role in regulating the anthocyanin biosynthetic pathway. Our previous study identified a homoeolog-specific copy, *MYB10-1B*, located on chromosome 1B, as a key regulator of fruit color. The natural mutation in *MYB10-1B*, such as in the variety ‘Florida Pearl’ leads to the development of white fruit. Building on this discovery, we applied CRISPR/Cas9-mediated homoeolog-specific editing to target the functional dominant allele, *MYB10-1B*, in the cultivated octoploid strawberry ‘Florida Brilliance’, successfully altering the fruit color from red to white. Gene expression analysis in the edited lines revealed downregulation of *MYB10-1B* and key anthocyanin biosynthesis genes (*CHS*, *DFR*, and *ANS*). Furthermore, whole-genome resequencing results showed precise on-target mutations in *MYB10-1B* with minimal off-target effects. This study highlights the successful application of homoeolog-specific CRISPR/Cas9-mediated gene editing in polyploid species and provides a foundation for functional genomics and advanced breeding strategies in strawberries. Importantly, our findings demonstrate that specific targeting of the dominantly expressed homoeologous copy is essential for inducing phenotypic changes in polyploids. This underscores the importance of precise gene editing in octoploid strawberry to drive trait improvement.

## Introduction

Strawberry (*Fragaria × ananassa* Duch.) is one of the most economically important fruit crops worldwide due to its attractive flavor, appealing appearance, and high nutritional value [1, 2]. Strawberry fruit color is a critical factor influencing consumer preference and represents an important trait in strawberry breeding programs [1, 3]. The cultivated strawberry exhibits a complex allo-octoploid genome (2*n* = 8*x* = 56), which has been challenging for breeding and genetic research [4, 5]. The first chromosome-scale reference genome of the cultivated octoploid strawberry ‘Camarosa’ was published in 2019 [5], proposing an evolutionary origin for its allo-octoploid genome derived from four diploid progenitors. This chromosome-scale assembly showed the presence of multiple homoeologous copies for most genes and introduced the concept of subgenome dominance, which refers to the unequal contribution of ancestral subgenomes to overall gene expression [5]. In recent years, advances in next-generation sequencing technologies, particularly the development of high-fidelity (HiFi) long-read sequencing, have enabled the generation of high-quality, haplotype-phased genome assemblies for octoploid strawberry cultivars. The phased genomes such as FaFB1 genome of ‘Florida Brilliance’ and FaRR1 of ‘Royal Royce’ provide unprecedented resolution, capturing the full allelic diversity and structural complexity of the octoploid strawberry genome [4, 6, 7]. These assemblies could significantly facilitate advanced genomic research and breeding of strawberries.

The characteristic red pigmentation of strawberry fruit is attributed to the accumulation of anthocyanins, a class of water-soluble pigments synthesized through the flavonoid biosynthetic pathway [3, 8]. Chalcone synthase (CHS) is the first enzyme in the flavonoid pathway, initiating a series of enzymatic reactions that lead to the production of anthocyanidins, the precursors to anthocyanins [3, 9]. Dihydroflavonol reductase (DFR) and anthocyanidin synthase (ANS) play a crucial role in generating the anthocyanin pigments that contribute to the unique color of strawberry fruit [8]. Pelargonidin derivatives were the dominant anthocyanins in strawberry fruits. Pelargonidin-3-glucoside accounted for ~80% of the total anthocyanins. DFR is known to preferentially use dihydrokaempferol as a substrate, which is a key factor in the synthesis of pelargonidin-derived anthocyanins. Combined enzyme assays of DFR and ANS using dihydrokaempferol have demonstrated the production of both pelargonidin and kaempferol. It has been described that the biosynthesis of anthocyanins in strawberries is regulated by *MYB10*, a member of a specific clade of R2R3 MYB transcription factors [10, 11]. In white-fruited strawberries, mutations in *MYB10* have been discovered in both diploid and octoploid strawberries [10, 12]. A single-nucleotide polymorphism (SNP, G–C), resulting in a nonsynonymous mutation in the *MYB10* gene of *Fragaria vesca*, was identified in white-fruited (yellow-fruited) diploid accessions such as ‘Yellow Wonder’, ‘Hawaii 4’, ‘White Soul’, ‘White Solemacher’, and ‘Pineapple Crush’, which alters a highly conserved tryptophan to serine within the DNA-binding domain [12]. An 8-bp insertion was identified in the white octoploid strawberry-specific allele *MYB10-2*, but not in the red octoploid strawberry-specific allele *MYB10-1*, resulting in the formation of a premature stop codon [9]. This mutation disrupts the function of gene and contributes to the white-color fruit phenotype. The *myb10*-*2* was found in several white-fruited octoploid cultivars such as ‘Snow Princess’ and ‘Florida Pearl’ [9, 13]. In the octoploid strawberry ‘Camarosa’, three *MYB10* homoeologous copies with full-length open reading frames were identified on subgenomes 1-1 (*MYB10-1*), 1-2 (*MYB10-2*), and 1-3 (*MYB10-3B*), but not on 1-4 [10]. Shortened *MYB10-2* promoter (*MYB10-2pro*) alleles were also identified in the white-fleshed octoploid accessions USA1, USA2, and FC157 [10]. The length of *MYB10-2pro* was 2.1 kb in USA1 and USA2, and 2.8 kb in FC157, which is dramatically shorter than the 23 kb *MYB10-2pro* in ‘Camarosa’. These truncated *MYB10-2pro* alleles could be candidates for the causal factor underlying the white-fleshed phenotype and may be useful for improving strawberry fruit color.

In octoploid strawberry, the use of multiple, often conflicting chromosome and subgenome nomenclature systems across studies has historically created confusion in comparative genomic research [10, 14]. Earlier assemblies, such as that of ‘Camarosa’, used a set of labels for chromosomes and homoeologs that were not consistently aligned with subgenomic origin. To address this issue, Hardigan *et al*. [14] proposed a unified nomenclature system based on phased, chromosome-scale haploid assemblies, which clarified subgenomic relationships and provided a systematic framework for naming chromosomes. This improved nomenclature was adopted in the recent ‘Royal Royce’ genome and subsequently implemented in the high-quality, haplotype-phased reference genome of ‘Florida Brilliance’ [4, 6]. To align with this standardized system and promote consistency within the strawberry research community, in this study, we designated the *MYB10* homoeologs in ‘Florida Brilliance’ according to their chromosomal and subgenomic locations: *MYB10-1B* (corresponding to *MYB10-2* in ‘Camarosa’), *MYB10-1C* (*MYB10-3B* in ‘Camarosa’), and *MYB10-1D* (*MYB10-1* in ‘Camarosa’). It is proposed that future studies adopt this updated nomenclature to ensure clarity, reproducibility, and effective communication in the field of strawberry genomics.

Clustered regularly interspaced short palindromic repeats (CRISPR)/CRISPR-associated (Cas) technology is a powerful tool for genome editing and has been used to improve crops [15]. Only a few CRISPR/Cas9 genome editing studies have been reported in octoploid strawberries [3, 16–18]. Knockout of *Reduced Anthocyanins in Petioles* (*RAP*) by CRISPR/Cas9-mediated mutagenesis resulted in white strawberry fruits in the octoploid strawberry ‘Ningyu’ [3]. RAP, involved in anthocyanin transport from the cytosol to the vacuole, regulated strawberry fruit coloration in an *MYB10*-independent manner. CRISPR/Cas9-mediated mutagenesis of *Tomato MADS box gene 6* (*TM6*) in the octoploid strawberry revealed that *FaTM6* plays an important role in flower development including anther development [16]. Petals in the mutant lines were smaller and greener at the preanthesis stage compared to the white petals of the wild type (WT). Additionally, anthers in the mutant lines were smaller and darker. Xing *et al*. [17] applied CRISPR/Cas9 to both diploid (*F. vesca*) and octoploid strawberries (*F*. × *ananassa*) via transient and stable transformation. A CRISPR/Cas9 vector was constructed with two single-guide RNAs (sgRNAs): one targeting *MYB10* and the other targeting *CHS*. Although delays in anthocyanin accumulation were not observed in strawberry fruits in octoploid strawberries, delays in anthocyanin accumulation were only partially observed in strawberry fruits in diploid strawberries.

Currently, CRISPR/Cas9-mediated gene editing in octoploid strawberries has relied on tissue culture methods, with no alternative yet established to bypass this step [3, 16, 18]. Establishing an efficient and genotype-independent tissue culture system in modern varieties is a primary obstacle to successful CRISPR/Cas9-mediated gene editing [19, 20]. Leaf discs of the octoploid strawberry cultivar ‘Camarosa’ [16] and leaf strips of ‘Ningyu’ [3] were used as explants for tissue culture. To establish a robust tissue culture process, our previous study optimized tissue culture conditions for ‘Florida Brilliance’ and Sweet Sensation® ‘FL127’ using runner and petiole segments. These optimized protocols have been successfully applied for CRISPR-mediated gene editing of *PDS* in cultivated octoploid strawberry [19]. As demonstrate in this study, all homoeologous copies of *PDS* were targeted because it is often challenging to specifically target a single homoeologous allele linked to a functional phenotypic change.

In this study, we explored the application of homoeolog-specific CRISPR/Cas9-mediated gene editing to target *MYB10-1B*, which is essential for anthocyanin biosynthesis and fruit coloration in the octoploid strawberry. Gene-edited lines with white fruit were successfully developed with a homozygous (or biallelic) mutation identified in *MYB10-1B*, but not in its homoeologs. This represents the importance of CRISPR/Cas9-guided homoeolog-specific gene editing for precisely modifying target breeding characteristics in cultivated octoploid strawberry.

## Results

### *MYB10* is the major factor in fruit coloration in octoploid strawberries

**Figure 1:**
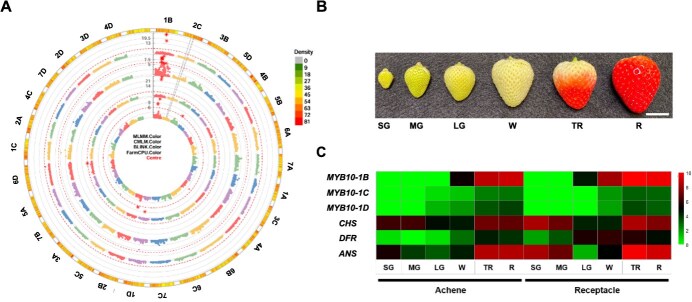
*MYB10*-*1B* is a candidate gene associated with white fruit color in octoploid strawberry. GWAS for white strawberry was conducted, resulting in circular Manhattan plots from the MLMM, CMLM, BLINK, and FarmCPU (A). Chromosome nomenclature based on the ‘Florida Brilliance’ genome is used instead of that of ‘Camarosa’ in the circular Manhattan plots. Gene expression patterns of *MYB10* and genes involved in anthocyanin biosynthesis pathway were investigated using RNA sequencing. Samples were collected at the Small Green (SG), Medium Green (MG), Large Green (LG), White (W), Turning Red (TR), and Red (R) stages (B). Gene expression levels were normalized to *z*-scores with log_2_(TPM + 1) for log-scale and visualized as a heatmap with a color scale (C). *ANS* (*Fxa5Bg634040*), anthocyanidin synthase; *CHS* (*Fxa7Ag324010*), chalcone synthase; *DFR* (*Fxa2Cg819580*), dihydroflavonol 4-reductase.

*MYB10* is the primary factor controlling fruit color variation in octoploid strawberries [10]. In our previous study, an 8-bp (ACTTATAC) insertion was identified in the open reading frame of *MYB10* in chromosome 1B (chromosome 1–2 for ‘Camarosa’ reference genome annotation), which causes the white fruit phenotype in strawberry [10]. A genome-wide association study (GWAS) with an F_1_ population derived from a biparental cross between red- and white-fruited parental lines showed SNPs associated with white fruit coloration. The majority of significant SNPs were detected in chromosome 1B across all different models (MLMM, CMLM, BLINK, and FarmCPU) tested (Fig. 1A). With the FarmCPU model, two significant SNPs (AX-184058011 and AX-184184084) were identified in chromosome 1B. It was found that an *MYB10* transcription factor gene, which is well known for its role in fruit coloration as the only gene strongly associated with anthocyanin biosynthesis in fruits, located ~370 kb downstream of the marker AX-184184084. The amplicon-sequencing data of *MYB10* from red- and white-fruited lines were mapped to the recently released high-quality haplotype-phased reference genome (‘Florida Brilliance’). The majority of reads (96.73%) were mapped to *MYB10-1B*, and 8-bp (ACTTATAC) insertion was only detected in the third exon of *MYB10* located in chromosome 1B (Fig. S1).

Furthermore, we analyzed transcriptome data from our previous study [21] to determine which *MYB10* homoeolog is predominantly expressed in achenes and/or receptacles of strawberry fruits. Transcriptome analysis revealed that *MYB10-1B* was highly expressed in turning and red achenes, as well as in white, turning, and red receptacles, compared to *MYB10-1C* and *MYB10-1D* (Fig. 1C). Our results are consistent with a previous finding [10], which demonstrated that among the *MYB10* homoeologs, *MYB10-1B* rather than *MYB10-1C* or *MYB10-1D* is the transcriptionally dominant form in ripening fruits. Taken together, we hypothesize that a knockout mutation in *MYB10-1B*, which is responsible for anthocyanin accumulation in ripening fruits, leads to the white fruit phenotype. In this study, CRISPR/Cas9-mediated mutagenesis was applied to one of the major commercial strawberry varieties ‘Florida Brilliance’ to knock out *MYB10-1B*.

### Gene structure and expression of homoeologous copies of *MYB10* in octoploid strawberry

**Figure 2:**
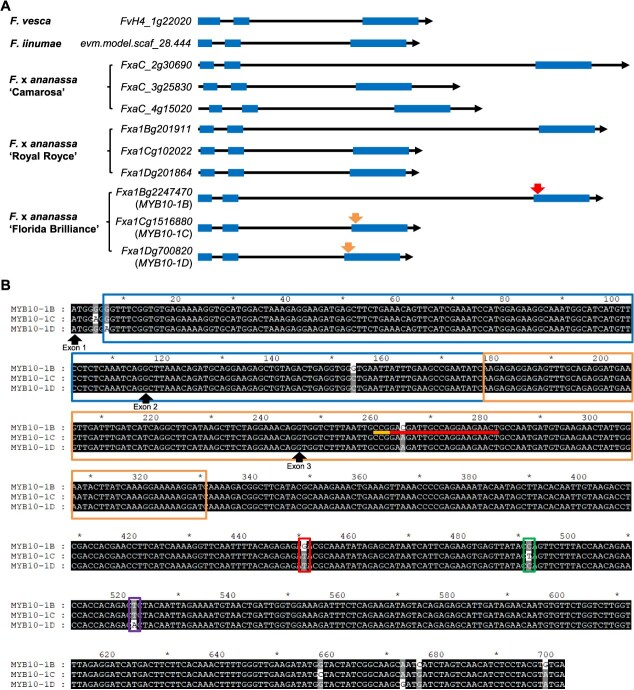
Gene structures of *MYB10* genes. *MYB10-1B*, *MYB10-1C*, and *MYB10-1D* indicate *Fxa1Bg2247470*, *Fxa1Cg1516880*, and *Fxa1Dg700820*, respectively. (A) A schematic diagram of *MYB10-1B*, its homoeologous genes, and orthologous genes. Exons are shown as boxes. The arrow in exon 3 of *MYB10-1B* indicates the on-target site, whereas the arrows in exon 3 of *MYB10-1C* and *MYB10-1D* indicate the off-target sites. (B) The CDSs of *MYB10-1B* and its homoeologous genes. The bars below exon 3 of *MYB10-1B* indicate the PAM sequence (short bar) and the guide sequences (long bar). Two MYB domains were identified in *MYB10*, and domain 1 and domain 2 are indicated by boxes. Boxes surrounding SNPs indicate positions specific to *MYB10-1B*, *MYB10-1C*, and *MYB10-1D*. Arrows indicate the first nucleotides of exon 1, exon 2, and exon 3 of *MYB10*-*1B*, *MYB10*-*1C*, and *MYB10*-*1D*.

*MYB10-1B*  (*Fxa1Bg1886930*) is 3202 bp long, with three exons encoding 233 amino acids (Fig. 2A). Using BLAST, homoeologous genes to *MYB10-1B* were identified as *Fxa1Cg1516880* (*MYB10-1C*) and *Fxa1Dg700820* (*MYB10-1D*) on chromosome 1C and 1D, respectively. There was no *MYB10* homoeolog detected on chromosome 1A. In ‘Florida Brilliance’, *MYB10* homoeologs were identified in three subgenomes, which is consistent with previous research on ‘Camarosa’ [10]. Similarly, three *MYB10* homoeologs were identified in the three subgenomes of ‘Royal Royce’ (Fig. 2A). The *MYB10* genes showed high levels of conservation in *F*. *vesca* and *F*. × *ananassa* species. In ‘Florida Brilliance’, *MYB10-1B* and its homoeologous genes exhibited high sequence homology in the coding regions (Fig. 2B). The protein structures of MYB10-1B, MYB10-1C, and MYB10-1D were predicted using RoseTTAFold from the Robetta server (https://robetta.bakerlab.org/) [22]. All three predicted proteins exhibited similar structural conformations (Fig. S2).

Guide sequence candidates targeting the exon regions of *MYB10-1B* were generated using Cas-Designer [23] with a focus on designing sequences that specifically target the MYB domain in *MYB10-1B* while minimizing off-target effects on homoeologous genes. This was achieved by carefully analyzing the positions of mismatches in the guide sequences. As the RuvC and HNH domains of the Cas9 protein cleave each DNA strand three nucleotides upstream of the NGG protospacer adjacent motif (PAM) [24], we manually selected a guide sequence that perfectly matched *MYB10-1B* but had a single mismatch at the third nucleotide from the PAM site in the homoeologous genes (Fig. 2B). This mismatch helped enhance specificity for *MYB10-1B*, reducing the potential for off-target activity. The JH1 entry vector was constructed to include the FveU6-2 promoter, which controls sgRNA transcription [25]. To finalize the guide sequence, a guanine was added to the 5′ end, resulting in a 21-nucleotide guide sequence (5′-gAGTTCTTCCTGGCAATCGTC). Additionally, an *MYB10-1B*-specific SNP (G in *MYB10-1B* and T in *MYB10-1C* and *MYB10-1D*), an *MYB10-1C*-specific SNP (T in *MYB10-1C* and C in *MYB10-1B* and *MYB10-1D*), and an *MYB10-1D*-specific SNP (A in *MYB10-1D* and T in *MYB10-1B* and *MYB10-1C*) were identified in exon 3 of the *MYB10* homoeologs (Fig. 2B). These subgenome-specific SNPs were used in Sanger sequencing to determine the specific subgenome in which CRISPR/Cas9 gene editing was generated.

### High-throughput screening to identify mutant plants using high-resolution melting analysis and amplicon sequencing

**Figure 3:**
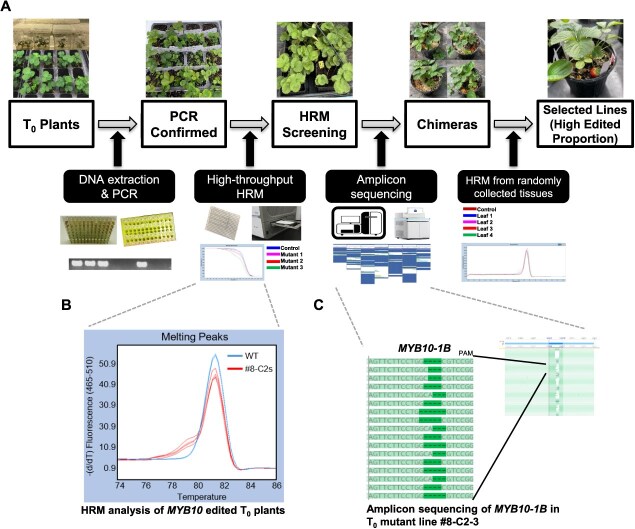
Workflow of high-throughput screening and identification of *MYB10*-*1B*-edited strawberry plants. (A) A schematic representation of high-throughput screening to identify mutant plants. Putative transgenic plants were PCR-confirmed, followed by HRM screening to detect mutations in *MYB10*. Mutant lines were further analyzed by amplicon sequencing to confirm and characterize mutation types. To evaluate mosaicism, HRM was performed on DNA extracted from multiple tissues (leaves or sepals) of the same T_0_ plants. Chimeric lines with higher proportions of edited cells were selected and advanced to the next generation. (B) Representative HRM melting curves of WT and *MYB10* edited T_0_ mutants. (C) Amplicon sequencing results of mutant line #8-C2-3, showing multiple types of insertions and deletions within the guide sequence target region of *MYB10-1B*. The sequence analysis program (Geneious) screenshot shows the diversity of mutation patterns in *MYB10-1B*.

Runner tips from ‘Florida Brilliance’ were used for tissue culture (Fig. S3) and transformed with *Agrobacterium* harboring a CRISPR/Cas9 vector. Transformed calli were screened using chemical selection with hygromycin and visual selection with green fluorescent protein (GFP). After regeneration, transgenic plants were confirmed by polymerase chain reaction (PCR) targeting the *Cas9* gene. To rapidly and cost-effectively screen *MYB10-1B* edited strawberry plants, we established a high-throughput screening system that combined high-resolution melting (HRM) analysis with targeted amplicon sequencing (Fig. 3A). All T_0_ transgenic plants were first subjected to HRM analysis to detect mutations within the *MYB10* target gene. Mutant plants were identified by HRM peaks showing bumps or tailing compared to the WT plants (Fig. 3B), while nonmutant plants displayed similar HRM patterns to the WTs. Based on the approach, mutation efficiencies (edited plants/total transgenic plants) were estimated at 29.63% (Table S1). Representative T_0_ mutant lines such as #6-C1-1 and #8-C2-3 were selected for amplicon sequencing to confirm the HRM results and characterize mutations in *MYB10* (Fig. S4). Sequencing reads were mapped to the ‘Florida Brilliance’ reference genome [6]. In the mutant line #6-C1-1, 12 464 reads were mapped to *MYB10-1B* with 2150 reads containing deletions or insertions, spanning at least 15 mutation types ranging from single base-pair deletions to 46-bp deletions (Fig. S4). By contrast, only 25 reads in *MYB10-1C* and 330 in *MYB10-1D* showed indels, indicating the strong specificity of editing at the *MYB10-1B* locus. In line #8-C2-3, 15 066 reads were mapped to *MYB10-1B* with ~1820 edited reads showing at least 20 different mutation types including deletions ranging from 1 to 69 bp (Fig. 3C and Fig. S4). Only a small fraction of reads from *MYB10-1C* and *MYB10-1D* showed indels. Similar results were obtained from other T_0_ lines subjected to amplicon sequencing, indicating that most HRM-positive plants were chimeric mutants containing multiple mutation types within *MYB10-1B*.

To better distinguish the extent of chimerism among T_0_ plants, we conducted a second round of HRM analysis using multiple tissue samples (4–6 leaves or sepals per plant) from selected lines (#6-C1-1, #6-C1-2, #8-C2-1, #8-C2-2, #8-C2-3, and #8-C2-4; Table S2). For example, #8-C2-2 and #8-C2-4 showed mutant-type HRM curves in three out of four leaves, while #8-C2-3 showed such peaks in two out of four leaves. Based on these results, #8-C2-2 and #8-C2-3 were identified as chimeric lines with relatively high proportions of edited cells and were selected for further propagation and advancement to the T_1_ generation.

### Alternation of strawberry fruit color by CRISPR/Cas9-mediated homoeolog-specific gene editing of *MYB10-1B*

**Figure 4:**
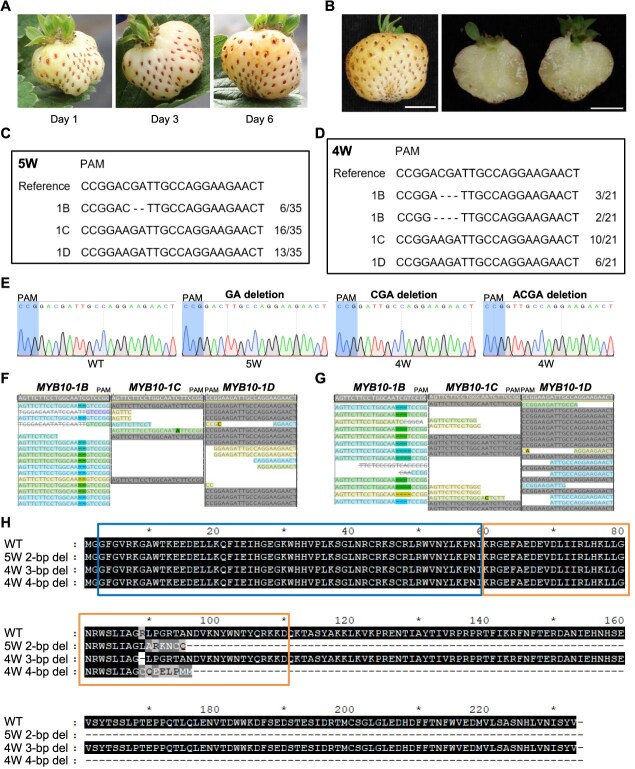
CRISPR/Cas9-mediated mutagenesis of *MYB10-1B* results in white strawberry fruits. (A) The white strawberries remained in the white stage for >6 days after the initial observation. (B) The skin and flesh of the white strawberry fruits retained white color, whereas the achenes changed to red. (C) A homozygous mutation in *MYB10-1B* was identified in the 5 W mutant line. The 2-bp (GA) deletions were identified in *MYB10-1B* by Sanger sequencing. The number of Sanger sequencing results showing the indicated sequence and the total number of results are indicated in the right corner. (D) A biallelic mutation in *MYB10-1B* was identified in the 4 W mutant line. The 3-bp (CGA) and 4-bp (ACGA) deletions were identified in *MYB10-1B* by Sanger sequencing. (E) Sanger sequencing chromatograms of the guide sequence target region of *MYB10-1B* in WT, 5 W, and 4 W. The two white strawberry lines were used for whole-genome resequencing. The reads were mapped to the ‘Florida Brilliance’ reference genome. Changes in sequences of *MYB10*-*1B* (on-target), *MYB10*-*1C* (off-target #1), and *MYB10*-*1D* (off-target #2) were investigated using Geneious Prime. A 2-bp (GA) deletion was identified in *MYB10-1B* in the 5 W mutant line (F). A 3-bp (CGA) deletion and a 4-bp (ACGA) deletion were identified in *MYB10-1B* in the 4 W mutant line (G). Few or no mutations were detected in *MYB10*-*1C* and *MYB10*-*1D* in both the 5 W and 4 W lines. (H) Amino acid sequences of *MYB10-1B* in gene-edited lines. The 2-bp deletion in the 5 W line and the 4-bp deletion in the 4 W line resulted in premature stop codons. The 3-bp deletion in the 4 W resulted in an arginine deletion. Protein domains in *MYB10-1B* predicted by InterProScan. Two MYB domains (IPR017930) were identified in *MYB10-1B*, and domain 1 and domain 2 are indicated by boxes.

A phenotyping-first approach was adopted to first evaluate strawberry fruit color and then investigate mutations at the on-target and off-target sites using white strawberries. Fruits were collected from T_0_ chimeric mutant lines and their runner-propagated plants. Achenes (seeds) were scarified with sulfuric acid and germinated on half-strength Murashige and Skoog (MS) medium supplemented with hygromycin (4 mg/l). A total of 13 T_1_ plants were used for phenotyping. Among the T_1_ plants derived from T_0_ #8-C2-3, three out of eight produced white strawberry fruits. Additionally, one out of five T_1_ plants derived from T_0_ #8-C2-2 produced nearly white with pale pink strawberries. The white strawberries collected from gene edited lines displayed typical characteristics of white strawberries (Fig. 4A and B, and Fig. S5), remaining white throughout development and not turning red. Both the skin and flesh of the fruit stayed white at full maturity, while the achenes turned red.

Whole-genome resequencing was conducted to detect mutations at the on-target and off-target candidate sites (Fig. 4, Fig. S6, and Table S3). Two T_1_ white-fruited lines (4 W and 5 W) and one T_1_ red-fruited line, all derived from the same T_0_ plant (#8-C2-3), were analyzed. In the 5 W mutant line, a homozygous 2-bp deletion (GA) was detected in *MYB10-1B* with few or no mutations observed in off-target candidates, including *MYB10-1C* and *MYB10-1D* (Fig. 4F and Fig. S6). In the 4 W mutant line, a biallelic mutation was identified in *MYB10-1B* consisting of a 3-bp deletion (CGA) and a 4-bp deletion (ACGA) (Fig. 4G and Fig. S6). Similarly, few or no mutations were detected in the off-target candidates. In the control line, few or no mutations were detected at both the on-target and off-target sites (Fig. S6). Although a few mutations were detected in the mapped reads from both the white-fruited mutant lines and the control, these were mostly nucleotide changes rather than deletions or insertions.

Using the *MYB10-1B*-specific guide sequence, mutations were successfully induced in *MYB10-1B* in the 5 W and 4 W mutant lines. Whole-genome resequencing of these mutant lines revealed that deletions occurred only in *MYB10-1B*, with no insertions or deletions detected in off-target candidate sites, including *MYB10-1C* and *MYB10-1D* (Fig. 4F and G, and Fig. S6). Sanger sequencing of the white fruit samples further cross-checked that mutations occurred only in *MYB10-1B* and not in its homoeologs (Fig. 4C–E), consistent with the whole-genome resequencing results. The 5 W line showed a homozygous mutation in *MYB10-1B* with no mutations identified in *MYB10-1C* or *MYB10-1D*. Similarly, the 4 W line exhibited a biallelic mutation in *MYB10-1B* with no mutations detected in *MYB10-1C* and *MYB10-1D*. The homozygous mutation in the 5 W line and the biallelic mutations in the 4 W line knocked out the function of the MYB10-1B transcription factor, resulting in the white fruit phenotype in both lines. Specifically, the 2-bp deletion in the 5 W line and the 4-bp deletion in the 4 W line caused frameshift mutations in *MYB10-1B*, resulting in premature stop codons (Fig. 4H). Additionally, the 3-bp deletion in the 4 W line led to the deletion of an arginine residue, which is located within the second MYB domain as predicted by InterProScan [26] (Fig. 4H). The deleted arginine residue may play a crucial role in the function of the MYB10-1B transcription factor or could affect protein folding and structure.

The occurrence of the 4 W and 5 W white-fruited mutant lines from a chimeric T_0_ #8-C2-3 mutant highlights the complexities of CRISPR/Cas9-mediated gene editing in polyploids. Amplicon sequencing of T_0_ #8-C2-3 seedling revealed >20 different mutation types in *MYB10-1B*, with the majority of mapped reads showing no mutations in the target region, indicating that chimeric nature of the T_0_ line (Fig. S4). This chimerism was further validated through HRM analysis of leaves collected from the mature T_0_ plant (Table S2), suggesting that not all the cells in the callus were edited prior to shoot organogenesis. As a result, only a portion of the cells carried the desired mutations, leading to the generation of both edited and nonedited sectors in the same plant. Despite this chimerism, the detection of *MYB10-1B* gene edited reads confirms successful transgene integration and the functional transcription of sgRNA driven by the U6 promoter and Cas9 driven by the UBQ promoter. The continued activity of Cas9 likely contributed to the ongoing induction of double-strand breaks (DSBs) in nonedited cells, even after organogenesis had occurred. This likely caused the nonedited cells in T_0_ #8-C2-3 to convert into edited cells, increasing the probability that gametes contained mutations. Through recombination of mutated gametes, homozygous mutations and biallelic mutations appear to have occurred in the 5 W (#8-C2-3-R1-3) and 4 W (#8-C2-3-R1-5) lines, respectively. Interestingly, although both lines originated from the same T_0_ #8-C2-3-R1 line, they exhibited different mutation types, with the 5 W line carrying a homozygous mutation and the 4 W line displaying a biallelic mutation (Fig. 4C and D). In contrast, the control line #8-C2-3-R1-4, which was derived from the same T_0_ #8-C2-3-R1 line, showed no insertions or deletions in *MYB10-1B* (Fig. S6).

### Effect of *MYB10-1B* mutations on gene expression in the anthocyanin biosynthesis in strawberry fruits

**Figure 5:**
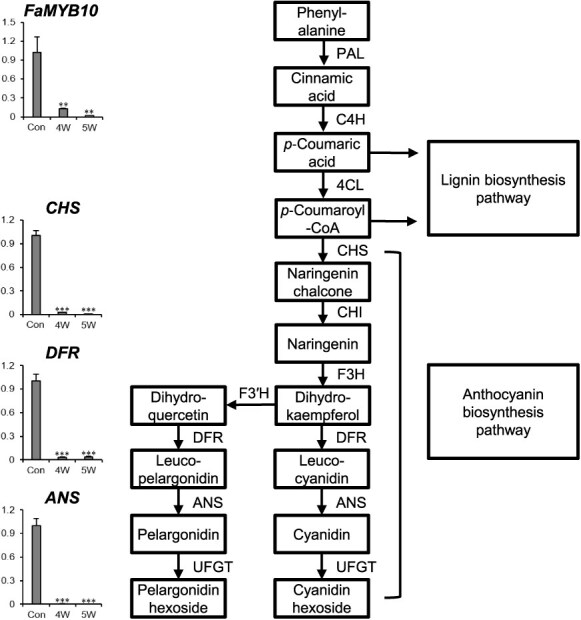
Mutations in *MYB10-1B* impacted the gene expression of *MYB10* and genes involved in the anthocyanin biosynthesis pathway in fruits, and the gene expression of *CHS* (*Fxa7Ag324010*), *DFR* (*Fxa2Cg819580*), and *ANS* (*Fxa5Bg634040*) in the mutant lines (4 W and 5 W) was analyzed using qRT-PCR and compared to the control (a sibling of 4 W and 5 W with red fruit), with asterisks indicating significant differences from the control according to the Student’s *t*-test (*P < 0.05, **P < 0.01, and ***P < 0.001); abbreviations: 4CL, 4-coumarate-CoA ligase; ANS, anthocyanidin synthase; C4H, cinnamic acid 4-hydroxylase; CHS, chalcone synthase; CHI, chalcone isomerase; DFR, dihydroflavonol 4-reductase; F3H, flavanone 3-hydroxylase; F3′H, flavonoid 3′-hydroxylase; PAL, phenylalanine ammonia lyase; UFGT, UDP-glucose:flavonoid 3-*O*-glucosyltransferase.

The mutations in *MYB10-1B* observed in the 5 W (homozygous) and 4 W (biallelic) mutant lines led to significant changes in the expression of *MYB10* and key genes involved in the anthocyanin biosynthesis pathway (Fig. 5). In the 5 W and 4 W lines, *MYB10* expression was significantly downregulated in white fruits compared to red fruits from the control, indicating a strong impact of the mutations on anthocyanin regulation. Additionally, *CHS* (*Fxa7Ag324010*), *DFR* (*Fxa2Cg819580*), and *ANS* (*Fxa5Bg634040*), involved in the anthocyanin biosynthesis pathway, were highly expressed in achenes and receptacles at the turning and red stages in WT (Fig. 1B and C). Gene expression analysis of the anthocyanin biosynthesis pathway revealed significant downregulation of key genes in the white fruits of both mutant lines. Specifically, *CHS* (*Fxa7Ag324010*), which encodes chalcone synthase, was significantly downregulated in white fruits from both 5 W and 4 W mutants, suggesting a disruption in the early steps of the pathway. *DFR* (*Fxa2Cg819580*), encoding dihydroflavonol 4-reductase, was also significantly downregulated, indicating a reduction in the conversion of dihydroflavonol to leucoanthocyanidin, a key intermediate in anthocyanin production. Additionally, *ANS* (*Fxa5Bg634040*), which encodes anthocyanidin synthase, showed significant downregulation in white fruits, further confirming that the mutations impacted the entire anthocyanin biosynthesis pathway. The results, as shown in Fig. 5, demonstrate that the mutations in *MYB10-1B* in the 5 W and 4 W lines not only affected *MYB10* expression but also had downstream effects on multiple genes critical for anthocyanin biosynthesis. These results are consistent with previous research on the white-fruited cultivar ‘Snow Princess’, which harbors an 8-bp insertion in white strawberry-specific allele *MYB10-2*, leading to significantly reduced expression of *MYB10*, *DFR*, and *ANS* at the overripe fruit stage [9]. To further clarify the contributions of different *MYB10* homeologs, we analyzed gene expression data from the red-fruited ‘FL13.65-160’ and the white-fruited ‘Florida Pearl’. Expression of *MYB10-1B* was consistently much higher in ‘FL13.65-160’, supporting its dominant role in anthocyanin biosynthesis, while *MYB10-1C* and *MYB10-1D* showed much lower expression and no compensatory upregulation in the white-fruited cultivar (Fig. S7). Overall, these findings highlight the critical role of *MYB10-1B* in activating anthocyanin biosynthesis during fruit ripening, with loss-of-function mutations in *MYB10-1B* leading to the white fruit phenotype.

## Discussion

CRISPR/Cas9 gene editing in octoploid strawberries, especially in the highly heterogeneous polyploid genome, presents unique challenges because of multiple homoeologs for each target gene [27]. Unlike diploid crops, where gene editing is more straightforward, the polyploid nature of strawberry requires precise targeting to avoid off-target effects [5, 28, 29]. In octoploid strawberries, the complexity of the polyploid genome poses major obstacles to achieving precise and efficient gene editing, which are critical for functional genomics and crop improvement. Fortunately, high-quality haplotype-phased reference genomes of octoploid strawberries, such as ‘Royal Royce’, ‘Florida Brilliance’, and ‘Yanli’ have recently been released [4, 6, 30]. High-quality reference genomes facilitate the identification of target genes and accurate homoeolog-specific sequences, allowing precise CRISPR/Cas9 gene editing in octoploid strawberries. This study is significant as it demonstrates the successful application of homoeolog-specific CRISPR/Cas9 gene editing in octoploid strawberries, specifically targeting *MYB10-1B*, a key regulator of anthocyanin biosynthesis, to produce white-fruited mutants. The ability to selectively target specific homoeologs without affecting closely related other genes would be important for advancing functional genomics and breeding efforts in strawberries.

*MYB10* plays a regulatory role in the phenylpropanoid pathway, including the anthocyanin biosynthesis pathway, during fruit and seed ripening, and is responsible for fruit and seed coloration in various plants [9, 31, 32]. In octoploid strawberry, *MYB10* is activated by ABA and is predominantly expressed in ripe and senescent fruits [11]. Genes involved in the phenylpropanoid pathway, such as *PAL*, *C4H*, *4CL*, *CHS*, *CHI*, *ANS*, *DFR*, and *UFGT*, were significantly downregulated in the white strawberry variety ‘Snow Princess’, which harbors an insertion mutation in *MYB10* [9], consistent with our results showing downregulation of *CHS*, *DFR*, and *ANS* in *MYB10-1B* gene-edited lines. Similar results have also been reported in the pale-yellow peach ‘Mochizuki’ [32]. The expression level of *PpMYB10.1* was higher in the red peach ‘Akatsuki’ than in ‘Mochizuki’. The gene expression of *CHS*, *F3H*, *DFR*, *ANS*, and *UFGT* was lower in ‘Mochizuki’ than in ‘Akatsuki’ after the initiation of red coloration in ‘Akatsuki’. In allo-hexaploid wheat, *TaMyb10-D* plays a predominant role in seed coloration compared to *TaMyb10-A* and *TaMyb10-B* [33]. In *TaMyb10-D* overexpression lines, *CHS*, *CHI*, *F3H*, *GT*, *DFR*, *ANS*, *F3′H*, and *UFGT* were upregulated compared to WT [31], which contrasts with our results showing downregulation of *CHS*, *DFR*, and *ANS* in *MYB10-1B* knockout lines. Due to its central role in regulating anthocyanin biosynthesis and its consistent association with fruit color across multiple plant species, *MYB10* represents a promising target for gene editing to modulate fruit pigmentation. Precise editing of functional copy of *MYB10* can be used not only to generate novel fruit color variants but also to accelerate trait-specific breeding in strawberry and other fruit crops.

Our gene expression data from this study (based on whole-fruit samples including receptacle and achenes, Fig. S7) and a previous study [10] indicate that the loss of *MYB10-1B* activity is specifically associated with the white receptacle phenotype. In our gene expression data analysis of the white-fruited cultivar ‘Florida Pearl’ and the red-fruited ‘FL13.65-160’, *MYB10-1B* was the dominantly expressed copy in both cultivars with much higher expression in ‘FL13.65-160’ whereas *MYB10-1C* and *MYB10-1D* were expressed at substantially lower levels. No increased expression of *MYB10-1C or MYB10-1D* was detected in ‘Florida Pearl’, suggesting that these homoeologs may not compensate for the loss of *MYB10-1B* (Fig. S7). This finding provides further evidence that *MYB10-1B* plays the predominant role in receptacle pigmentation, while red pigmentation in achenes may be controlled by partially independent regulatory pathways. As shown in Fig. S7A, the visible difference between the two varieties is fruit flesh color (white vs red), while both have red in achenes. Our gene expression analysis also revealed no significant differences in the expression of the *MYB10-1C* and *MYB10-1D* homoeologs, whereas *MYB10-1B* showed a clear difference between the two fruit types. This suggests that *MYB10-1B* is the major determinant of receptacle color, whereas achene color is regulated through a genetically distinct anthocyanin pathway. This finding supports previous studies highlighting the unique regulatory roles of achenes in strawberry development [34, 35]. For instance, Zhongchi Liu’s group demonstrated that achenes act as the ‘true fruits’ and regulate receptacle growth via auxin signaling [34, 35]. A previous study also provides evidence for the role of the *MYB10-1B* allele as the primary factor of fruit receptacle color [10]. It shows that the expression of this specific allele *MYB10-2* (*MYB10-1B*) is highly correlated with red fruit color, while other *MYB10* homoeologs do not show the same strong correlation. This would directly support our finding that *MYB10-1B* is the key regulator for receptacle pigmentation.

The design of guide sequences and the selection of target DNA sites are critical steps in CRISPR/Cas9 gene editing, as they directly influence the specificity and efficiency of the editing process [36, 37]. The seed region, which consists of 10 nucleotides adjacent to the PAM in the target DNA, plays a key role in determining the cleavage efficiency of the Cas9 protein [38]. Mismatches in the seed sequence region have a more significant impact on CRISPR/Cas9 activity than mismatches in nonseed sequences, with Cas9 showing less tolerance to mismatches in the seed sequence. The position of these mismatches can also affect the specificity of the editing process. Off-targeted prediction, as measured by the cutting frequency determination score, considers penalties for various types of mismatches within the guide sequence [39]. For this study, a guide sequence was selected that perfectly matches *MYB10-1B* but contains a single mismatch within the seed sequence of *MYB10-1C* and *MYB10-1D*. The mismatch occurs at the third nucleotide upstream from the PAM site, where Cas9 cleaves [24]. Additionally, since the U6 promoter requires a G at the 5′ end for efficient transcription of sgRNA [40], an extra G was added at the 5′ end of the guide sequence instead of replacing the existing nucleotide [41]. This adjustment may enhance the specificity of *sp*Cas9 by reducing unwinding promiscuity [42]. It has been shown that a single mismatch in the seed sequence prevented CRISPR/Cas9-mediated mutagenesis in the octoploid ‘Camarosa’ strawberry [16]. Specifically, a mismatch between sgRNA1 and allele #5 of *FaTM6* at the fourth nucleotide from the PAM site prevented gene editing, while the other four *FaTM6* alleles were successfully edited. Similarly, in tomato, a single mismatch 11 nucleotides upstream from the PAM influenced Cas9 tolerance [43]. A guide sequence perfectly matched to *Solyc01g066970* but containing a single mismatch to *Solyc01g066950* led to mutations in both genes in some mutant lines, but in others, only *Solyc01g066970* was mutated. In this study, the mismatch at the third nucleotide from the PAM in the seed sequence likely explains the absence or reduction of mutations in *MYB10-1C* and *MYB10-1D* that ensure specificity to *MYB10-1B*.

Chimeric mutant lines created by CRISPR/Cas9 are often reported in strawberries [16, 25]. In our study, we induced the 4 W and 5 W white-fruited mutant lines from the chimeric T_0_ #8-C2-3 mutant line. Similar findings were reported in diploid *F*. *vesca*, where a predicted chimeric mutant plant produced five different types of *FveARF8-*edited plants and one nonedited plant in the next generation [25]. The T_0_ JH19-ARF8-66 plant was initially identified as a heterozygous 4-bp deletion mutant line, but most of the 10 T_1_ progeny exhibited either heterozygous or homozygous 2-bp deletions in *FveARF8*, while one progeny showed no mutation. The results closely resemble our findings, where a single T_0_ plant produced various T_1_ gene-edited and nonedited plants. Similarly, chimeric mutant lines induced by CRISPR/Cas9 gene editing have also been reported in the octoploid ‘Camarosa’ [16]. Nine, 7, and 10 different alleles of *FaTM6* were detected by amplicon sequencing in the *tm6-1*, *tm6-7*, and *tm6-9*, respectively. The *tm6-1* and *tm6-9* lines are considered chimeric mutant lines due to the presence of more than eight alleles.

In this study, we applied a robust screening of mutants by HRM analysis and further conducted amplicon sequencing for the HRM-screened mutants (Fig. 3 and Fig. S4). However, all the lines subjected to amplicon sequencing in our study were found to be chimeric mutant lines. While HRM analysis allowed for a rapid and cost-effective method for screening a large number of CRISPR/Cas9-induced mutants, it also detected chimeric mutants due to its high resolution of HRM [44, 45]. After initial screening with HRM, it is necessary to identify the mutations generated in each homoeolog and exclude any suspected chimeric mutants. If homoeolog-specific primers were available, Sanger sequencing using PCR products could be a practical alternative to amplicon sequencing. Tools like ICE [46] could further assist in identifying and quantifying insertions and deletions based on Sanger sequencing data, providing a valuable resource for interpreting the genetic modifications. Sanger sequencing using PCR products can save time and reduce costs compared to amplicon sequencing. Moreover, we used the high-quality reference genome of the cultivar employed for gene editing in amplicon sequencing to obtain accurate mapping results. If a variety with a different genetic background from the cultivar used to create the reference genome is utilized for gene editing, the reads may not map accurately to each homoeolog.

In conclusion, this study demonstrates the successful application of homoeolog-specific CRISPR/Cas9 gene editing in octoploid strawberry by precisely targeting *MYB10-1B* to generate a stable white fruit phenotype. The guide sequence was designed to effectively target *MYB10-1B* while minimizing off-target effects on closely related homoeologs *MYB10-1C* and *MYB10-1D*. Knockout of *MYB10-1B* led to the downregulation of key anthocyanin biosynthesis genes such as *CHS*, *DFR*, and *ANS* in ripened fruits, confirming its functional role in fruit coloration. Our findings highlight that homoeolog-specific genome editing could offer a promising approach to broaden fruit quality traits and support the advancement of strawberry breeding.

## Materials and methods

### Plant material

The commercial octoploid strawberry cultivar ‘Florida Brilliance’ was grown in a greenhouse at the Gulf Coast Research and Education Center in Balm, FL, USA (27°75′ N, 82°22′ E). To advance transgenic plants, achenes (seeds) were harvested from T_0_ transgenic plants. Completely dry T_1_ achenes were subjected to sulfuric acid scarification for 10 min, ensuring complete immersion. After scarification, the achenes were rinsed thoroughly with deionized water 10 times. The achenes were dried on paper towels for at least 2 days. The dried achenes were then placed on half-strength MS medium supplemented with hygromycin (4 mg/l) in Petri dishes, and incubated at 23°C. Surviving seedlings were transplanted into soil and grown in the greenhouse.

### GWAS analysis of fruit color

GWAS was conducted using the genotype and phenotype data from Castillejo *et al*. [10]. A total of 95 strawberry plants from the UF family 17.66 were evaluated for fruit color, and genotype data were collected using the FanaSNP 50 K Array [47]. In this study, GWAS was performed in R using GAPIT with the MLMM, CMLM, BLINK, and FarmCPU models [48–51].

### Gene expression analysis of *MYB10* homeologs during strawberry fruit development

To evaluate the gene expression levels of *MYB10-1B* and its homoeologs during fruit development, transcriptome data from our previous study [21] were utilized. Achenes and receptacles were collected at the Small Green (SG), Medium Green (MG), Large Green (LG), White (W), Turning Red (TR), and Red (R) stages. The gene expression levels of *MYB10-1B*, *MYB10-1C*, and *MYB10-1D* were visualized as a heatmap plot. Transcriptome data analysis was performed as described in Jang *et al*. [21].

### MYB10 protein structure prediction

The 3D structures of MYB10-1B, MYB10-1C, and MYB10-1D were modeled using an *ab initio* approach with RosettaFold from the Robetta server (https://robetta.bakerlab.org/) [22]. The most reliable 3D structure for each MYB10 protein was selected based on the confidence value.

### Construction of CRISPR/Cas9 vector

Guide sequences targeting *MYB10-1B* was designed using the CRISPR RGEN Tools [23]. A guanine was added to the 5′ end of the selected guide sequence. Potential off-targets with up to four mismatches were predicted using Cas-OFFinder [52]. A 21-bp guide sequence was used for CRISPR/Cas9 vector construction as described in Zhou *et al.* [25]. The guide sequence was introduced into the JH1 entry vector and subsequently into the JH19 destination vector. The CRISPR/Cas9 vector was inserted into the *Agrobacterium tumefaciens* (EHA105) using a freeze–thaw method [53].

### Strawberry tissue culture and *Agrobacterium*-mediated transformation of octoploid strawberry

Strawberry tissue culture was conducted following Kim *et al*. [19] with some modifications. Runners were cut 4- to 7-cm long from the runner tip. Runners were surface-sterilized with 70% ethanol for 10 min, rinsed with distilled water for 3–5 min three times, further sterilized with 10% commercial Clorox and 0.25% Tween 20 for 10 min, and rinsed with distilled water three times. The runners were placed on autoclaved filter paper to dry, then cut into 0.8- to 1-cm segments with a surgical blade. The explants were transferred to the SRM11, incubated for 1 day, and used for *Agrobacterium*-mediated transformation. The explants were incubated in the *Agrobacterium* culture for 10 min with occasional agitation, placed on autoclaved filter paper to remove *Agrobacterium*, and then cocultured for 2 days at 24 ± 2°C under dark conditions. The explants were transferred to the SRM11 with 150 mg/l timentin, 150 mg/l cefotaxime, and 2–4 mg/l hygromycin. Explants were transferred to fresh SRM11 with antibiotics every 2 weeks for 8–12 weeks until shoot initiation. Transgenic calli were screened by GFP signal detected using the Olympus SZX2-ILLT microscope (Olympus Corp., Tokyo, Japan) as described in Kim *et al*. [19]. Calli were transferred to the EM11, while 150 mg/l timentin, 150 mg/l cefotaxime, and 2–4 mg/l hygromycin were supplemented to the EM11 in this study. Calli were transferred to fresh EM11 with antibiotics every 3 weeks for 3–6 weeks until shoots reach 2–3 cm with two to three leaves. The HF was used to facilitate rooting. Individual shoots were transferred to the HF with antibiotics. Individual well-rooted seedlings were transferred to the soil. After 2 weeks of acclimation in a growth room, the seedlings were transferred to a greenhouse.

### Genomic DNA extraction

Genomic DNA was extracted from fruits and leaves using the cetyltrimethylammonium bromide method as described by Oh *et al*. [54]. For leaf samples, young leaves were collected and frozen in liquid nitrogen. For fruit samples, fully ripened fruits were collected and frozen in liquid nitrogen. Each sample was ground into a fine powder using a Fisher Scientific™ PowerGen™ High-Throughput Homogenizer (Thermo Fisher Scientific Inc., DE, USA). Genomic DNA was extracted using each ground sample.

### High-resolution melting assay

HRM was conducted to identify mutations in *MYB10*. HRM experiment and HRM data analysis were performed as described in Castillejo *et al.* [10]. The sequences of *MYB10*-specific primers for HRM are provided in Table S4. HRM analysis using ‘Florida Pearl’ and ‘Florida Brilliance’ was performed with the UFWsHRM01 marker reported by Jang *et al.* [55].

### Amplicon sequencing

The amplicon sequencing raw data used in Castillejo *et al.* [10] were mapped to the ‘Florida Brilliance’ reference genome [6]. The data were obtained from white and red fruit accessions derived from the UF family 17.66 [10]. RNA was extracted from each sample, and a cDNA pool consisting of white fruits or red fruits was used for amplicon sequencing. The amplicon-sequencing raw data of white fruits were used for trimming using Trimmomatic [56]. Clean reads were mapped to the octoploid strawberry ‘Florida Brilliance’ reference genome (https://www.rosaceae.org/Analysis/14031408) using BWA (alignment via Burrows–Wheeler transformation, version 0.7.8-r455) [57]. The sequence alignment/map format was sorted and converted to binary alignment/map (BAM) format using Samtools ver. 0.18 [58]. Only qualified alignments (mapping quality >30) were stored in each BAM file and visualized using Geneious Prime ver. 2023.0.4.

Furthermore, mutants identified by HRM were used for amplicon sequencing to investigate nucleotide sequences in homoeologous copies of *MYB10* on subgenomes 1B, 1C, and 1D. The primer sequences are listed in Table S4. PCR products were used for amplicon sequencing as described in Castillejo *et al.* [10]. Reads were trimmed, mapped to ‘Florida Brilliance’ reference genome, and visualized as described as above.

### Whole-genome resequencing

Whole-genome resequencing was conducted to investigate on-target and off-targets. Genomic DNA was extracted from the leaves of #8-C2-3-R1-3 (5 W), #8-C2-3-R1-5 (4 W), and #8-C2-3-R1-4 (red fruit control). Library construction and sequencing were performed by Novogene (Novogene Corp., Beijing, China). The genomic DNA was randomly sheared into small fragments. The sheared fragments were end-repaired, A-tailed, and used for ligation with Illumina adapters. The adapter-ligated fragments were PCR-amplified, selected based on size, and purified. Libraries were pooled and sequenced on the Illumina Novaseq platforms (Illumina Inc., CA, USA). The low-quality reads and adapter sequences were trimmed, mapped to the ‘Florida Brilliance’ reference genome [6], and visualized using Geneious Prime ver. 2023.0.4.

### Nested PCR

Nested PCR was conducted to investigate mutations in *MYB10-1B* and its homoeologous genes in *MYB10-1B*-edited plants. Primers for nested PCR were designed using the conserved region of *MYB10-1B* and its homoeologous genes. The list of primers for nested PCR is available in Table S4. An initial round of nested PCR was performed using TaKaRa Ex Taq (Takara Bio, CA, USA) under the following conditions: 98°C for 5 min, followed by 35 cycles of 98°C for 30 s, 52°C for 30 s, and 72°C for 1 min, with a final extension at 72°C for 5 min. A second round of nested PCR was performed with minor modifications compared to the initial round. Half amount of MgCl_2_ was used for the PCR reaction preparation, and the annealing temperature was adjusted to 56°C. PCR products were purified by gel elution using Expin Gel SV (GeneAll Biotechnology, Seoul, Republic of Korea), ligated with the pLUG-Prime TA-cloning vector (iNtRON Biotechnology, Gyeonggi-do, Republic of Korea), and used for Sanger sequencing.

### RNA extraction and quantitative reverse transcription PCR

RNA extraction and quantitative reverse transcription PCR (qRT-PCR) were performed as described by Lee *et al*. [59], with some modifications. Ripened strawberry fruits collected were frozen in liquid nitrogen. A total of 150 mg of fine powder sample was used for RNA extraction. For each sample, 1 μg of total RNA was treated with DNase I (Invitrogen, MA, USA) to remove genomic DNA contamination, following the manufacturer’s protocol. The treated RNA was then reverse-transcribed to cDNA using oligo (dT) primers and M-MLV reverse transcriptase (M0253, New England Biolabs, MA, USA).

To validate the gene expression associated with anthocyanin biosynthesis in fruit, qRT-PCR was carried out with the *FaGAPDH* gene serving as the housekeeping control. The primer sequences are listed in Table S4. PCR conditions were set at 95°C for 5 min, followed by 40 cycles of 95°C for 20 s, 60°C for 20 s, and 72°C for 20 s. The qRT-PCR was performed in triplicate for technical replicates.

### RNA-seq analysis of MYB10 expression in octoploid strawberry

To assess *MYB10* expression associated with fruit color variation, whole-fruit samples (including receptacle and achenes) were collected from two octoploid strawberry varieties: the elite breeding line ‘FL 13.65-160’ (pinkish-red fruit) and the white-fruited cultivar ‘Florida Pearl’. The ‘FL 13.65-160’ line was developed in 2013, and it was later used as one of the progenitors in the development of ‘Florida Pearl’. Approximately, 10 ripe fruits were harvested from each variety, pooled, and used for RNA extraction using Spectrum™ Plant Total RNA Kit (Sigma-Aldrich, MO, USA). Sequencing libraries were prepared following the Illumina protocol and sequenced as paired-end reads (2 × 150 bp) on an Illumina NovaSeq platform. Raw reads were filtered and trimmed using Trimmomatic (v0.16), and read quality was evaluated with FastQC (https://www.bioinformatics.babraham.ac.uk/projects/fastqc/). Clean reads were mapped to the *F.* × *ananassa* reference genome (‘Camarosa’ v1.0) using HISAT2 (v2.14) with default parameters. Gene-level read counts were obtained using featureCounts (Subread v2.0) based on the corresponding gene annotations. Total read counts were normalized for sequencing depth, and transcripts per million (TPM) values were calculated by accounting for both gene length and library size.

## Supplementary Material

Web_Material_uhaf272
