# Supplementary material for: Whitening fruit by CRISPR/Cas9-mediated homoeolog-specific gene editing of MYB10-1B in strawberry (F. × ananassa)
Source: Hortic Res. 2025 Oct 15;13(1):uhaf272. doi: 10.1093/hr/uhaf272 (PMC12863208; doi:10.1093/hr/uhaf272)
Supplement: Web_Material_uhaf272 [file web_material_uhaf272.zip › Supplementary Figure 1.pptx]

## Slide 1
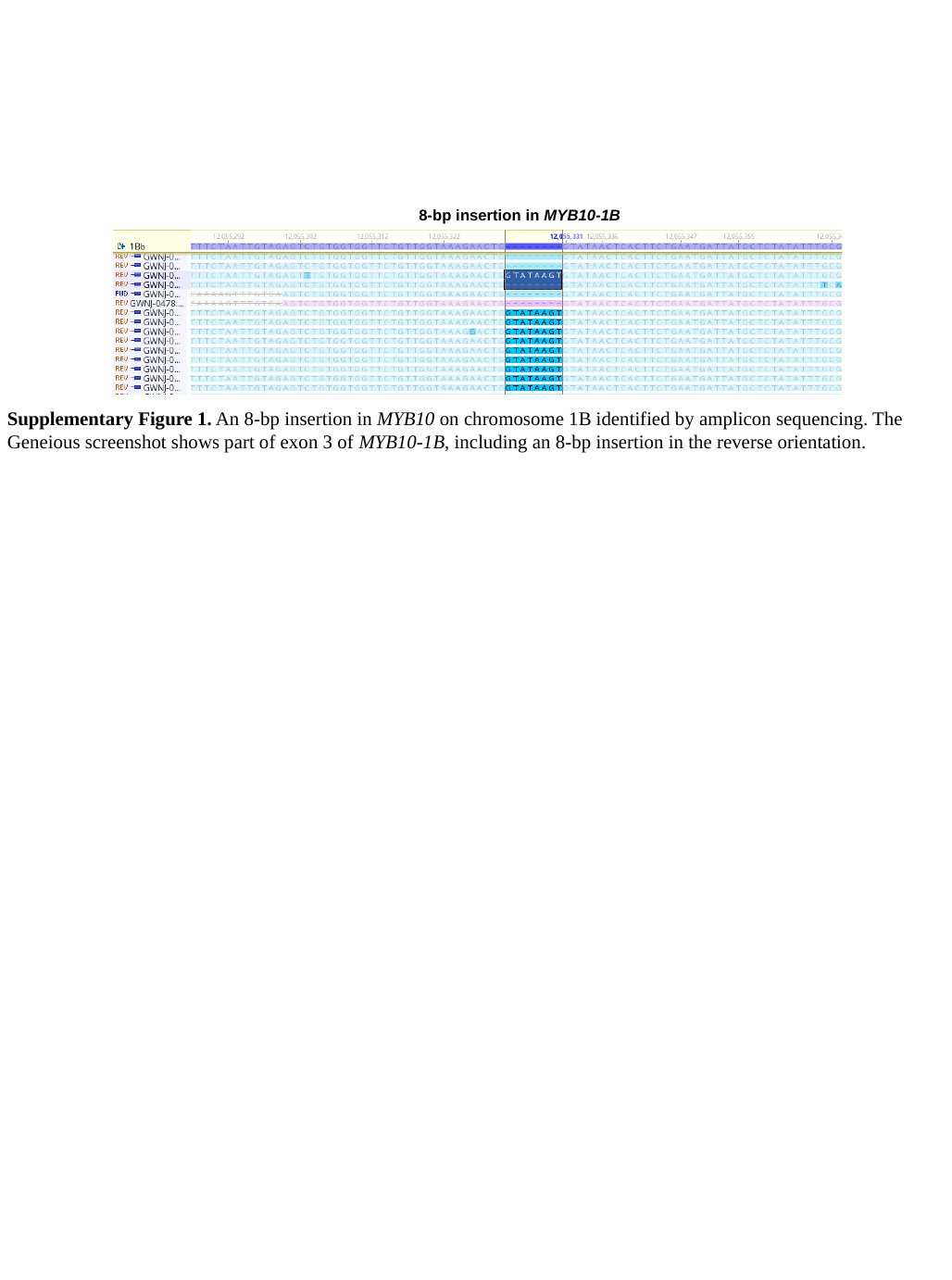

8-bp insertion in MYB10-1B
Supplementary Figure 1. An 8-bp insertion in MYB10 on chromosome 1B identified by amplicon sequencing. The Geneious screenshot shows part of exon 3 of MYB10-1B, including an 8-bp insertion in the reverse orientation.
