# Supplementary material for: Whitening fruit by CRISPR/Cas9-mediated homoeolog-specific gene editing of MYB10-1B in strawberry (F. × ananassa)
Source: Hortic Res. 2025 Oct 15;13(1):uhaf272. doi: 10.1093/hr/uhaf272 (PMC12863208; doi:10.1093/hr/uhaf272)
Supplement: Web_Material_uhaf272 [file web_material_uhaf272.zip › Supplementary Figure 2.pptx]

## Slide 1
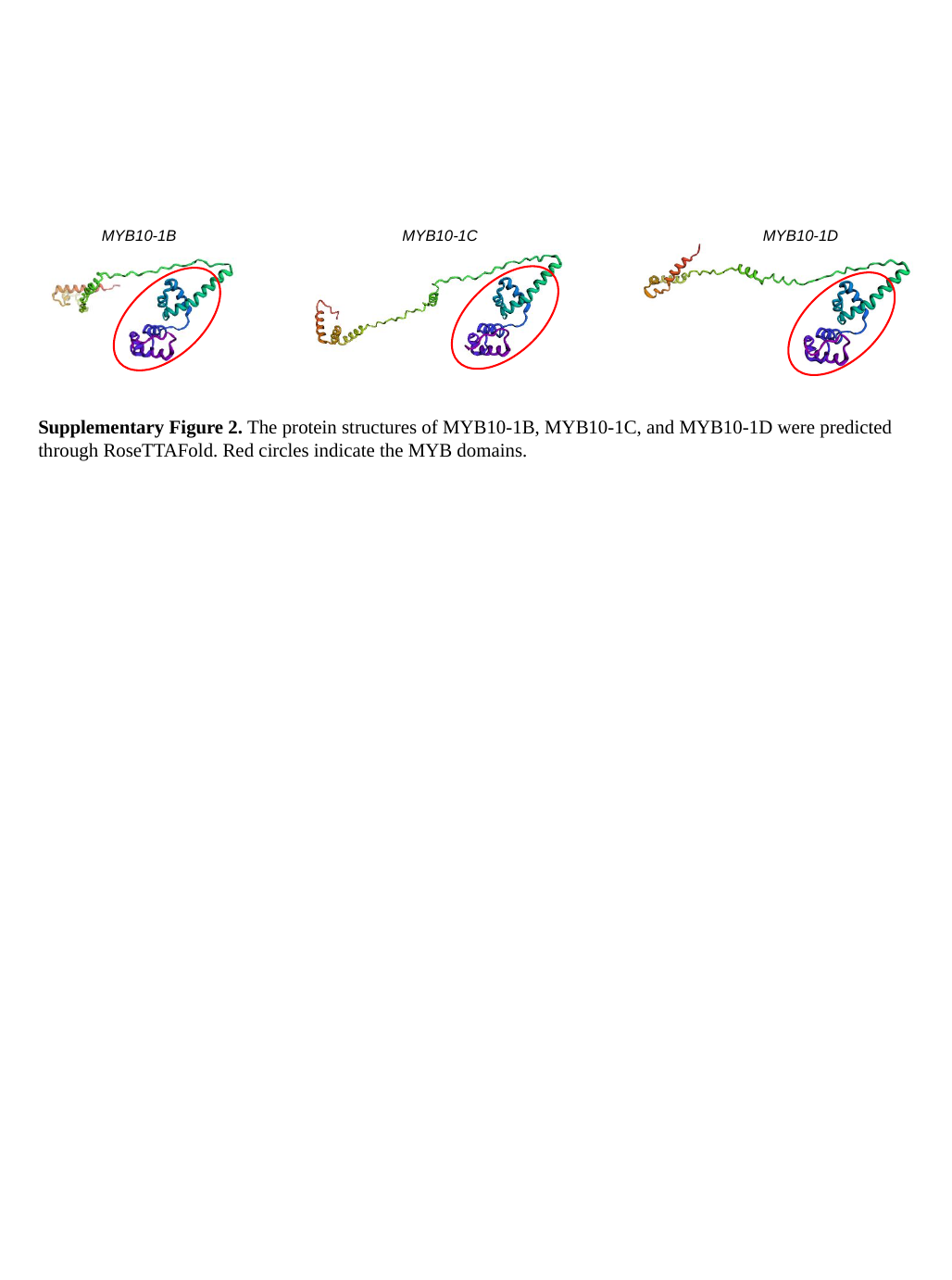

MYB10-1B
MYB10-1C
MYB10-1D
Supplementary Figure 2. The protein structures of MYB10-1B, MYB10-1C, and MYB10-1D were predicted through RoseTTAFold. Red circles indicate the MYB domains.
