# Supplementary material for: Whitening fruit by CRISPR/Cas9-mediated homoeolog-specific gene editing of MYB10-1B in strawberry (F. × ananassa)
Source: Hortic Res. 2025 Oct 15;13(1):uhaf272. doi: 10.1093/hr/uhaf272 (PMC12863208; doi:10.1093/hr/uhaf272)
Supplement: Web_Material_uhaf272 [file web_material_uhaf272.zip › Supplementary Figure 3.pptx]

## Slide 1
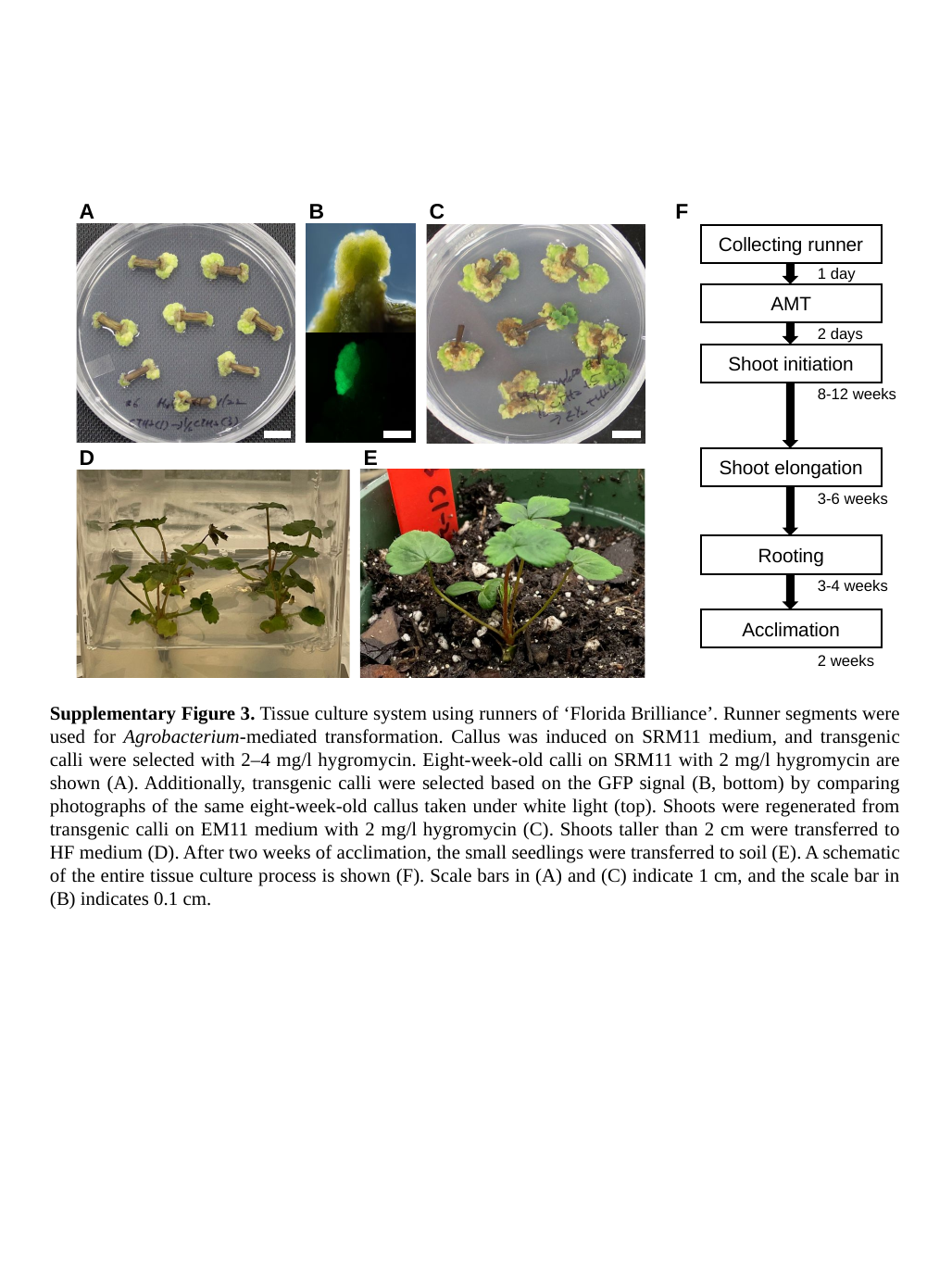

A
B
C
F
Collecting runner
1 day
AMT
2 days
Shoot initiation
8-12 weeks
D
E
Shoot elongation
3-6 weeks
Rooting
3-4 weeks
Acclimation
2 weeks
Supplementary Figure 3. Tissue culture system using runners of ‘Florida Brilliance’. Runner segments were used for Agrobacterium-mediated transformation. Callus was induced on SRM11 medium, and transgenic calli were selected with 2–4 mg/l hygromycin. Eight-week-old calli on SRM11 with 2 mg/l hygromycin are shown (A). Additionally, transgenic calli were selected based on the GFP signal (B, bottom) by comparing photographs of the same eight-week-old callus taken under white light (top). Shoots were regenerated from transgenic calli on EM11 medium with 2 mg/l hygromycin (C). Shoots taller than 2 cm were transferred to HF medium (D). After two weeks of acclimation, the small seedlings were transferred to soil (E). A schematic of the entire tissue culture process is shown (F). Scale bars in (A) and (C) indicate 1 cm, and the scale bar in (B) indicates 0.1 cm.
