# Supplementary material for: Whitening fruit by CRISPR/Cas9-mediated homoeolog-specific gene editing of MYB10-1B in strawberry (F. × ananassa)
Source: Hortic Res. 2025 Oct 15;13(1):uhaf272. doi: 10.1093/hr/uhaf272 (PMC12863208; doi:10.1093/hr/uhaf272)
Supplement: Web_Material_uhaf272 [file web_material_uhaf272.zip › Supplementary Figure 4.pptx]

## Slide 1
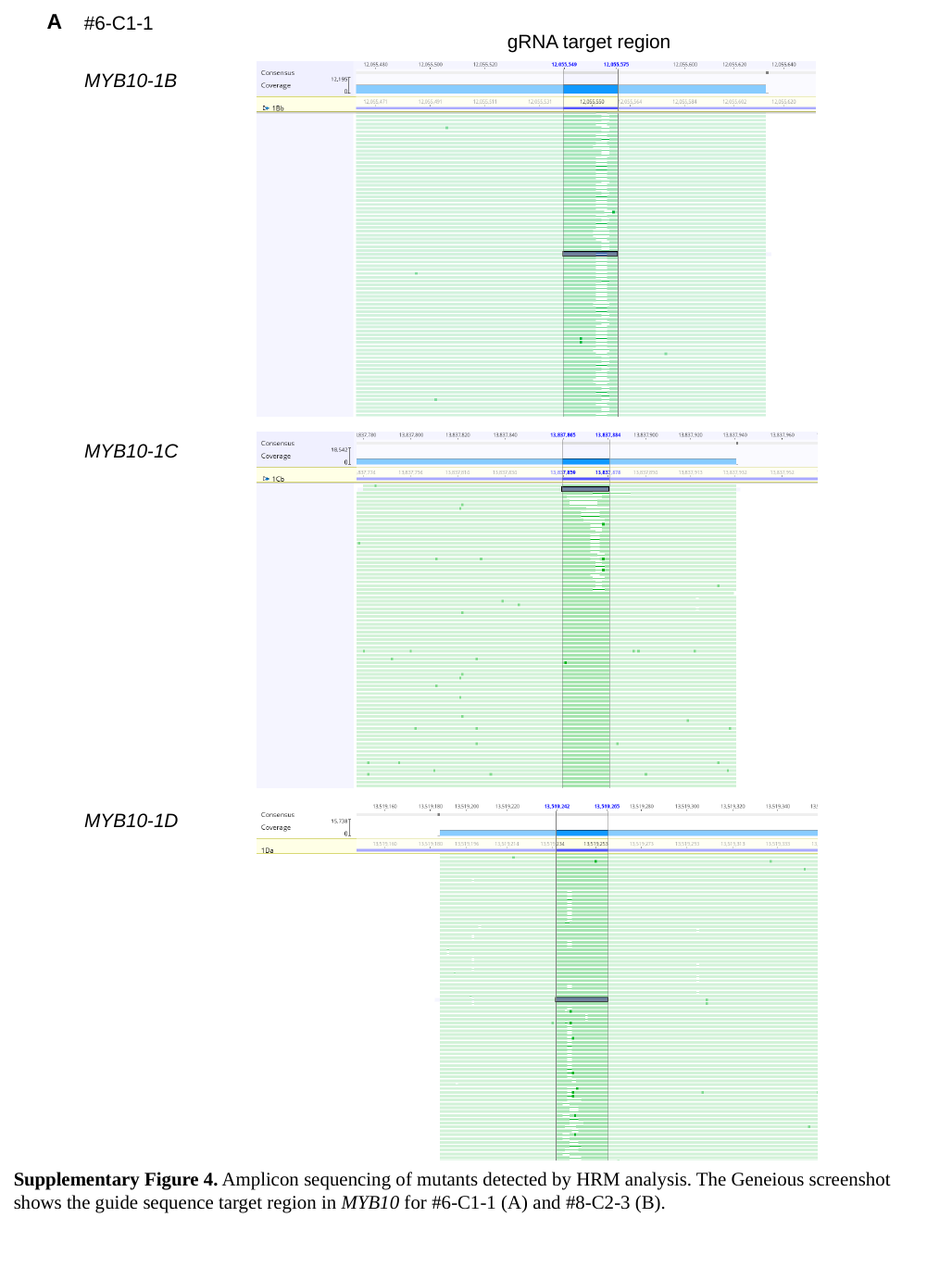

A
#6-C1-1
gRNA target region
MYB10-1B
MYB10-1C
MYB10-1D
Supplementary Figure 4. Amplicon sequencing of mutants detected by HRM analysis. The Geneious screenshot shows the guide sequence target region in MYB10 for #6-C1-1 (A) and #8-C2-3 (B).

## Slide 2
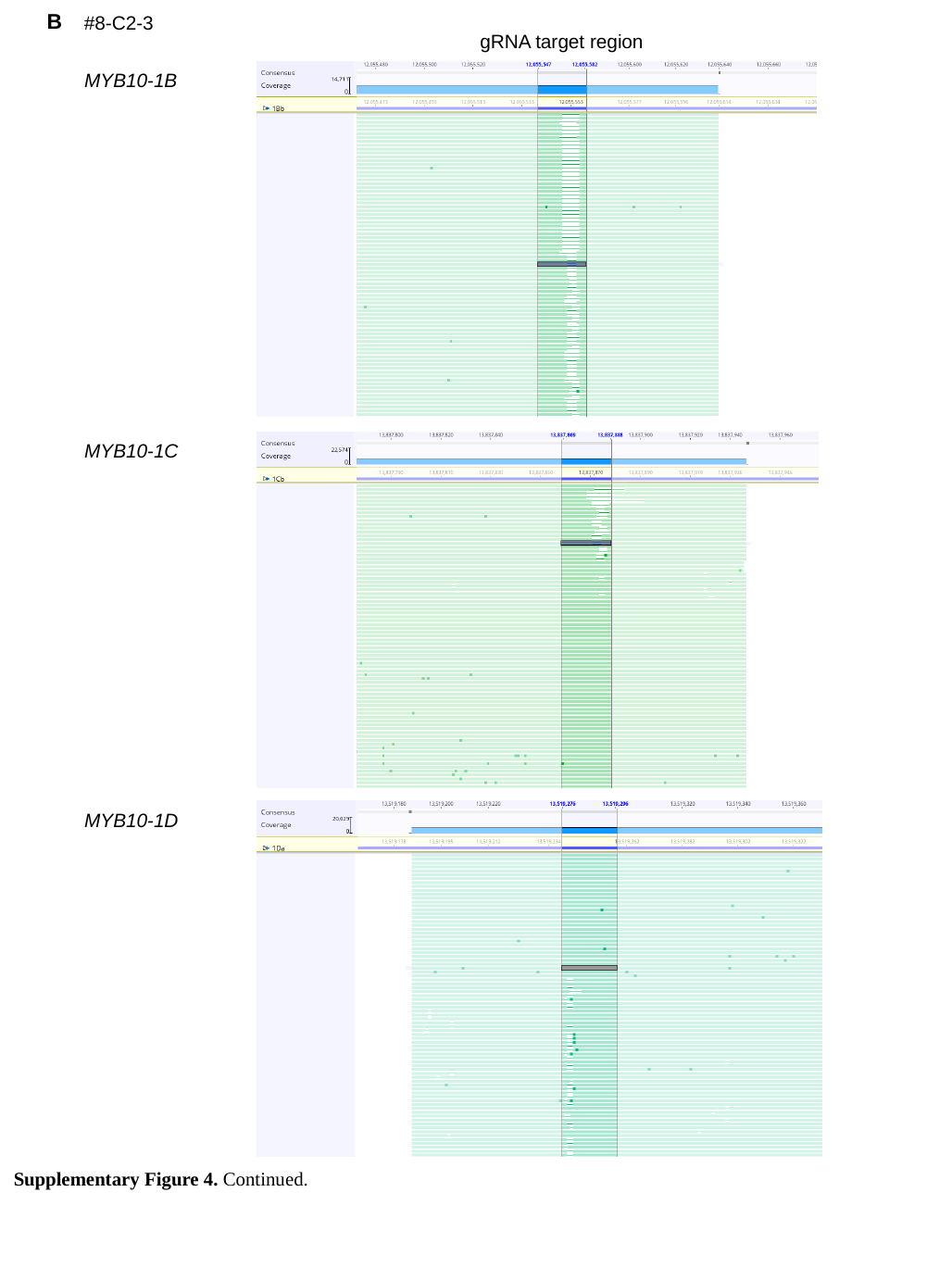

B
#8-C2-3
gRNA target region
MYB10-1B
MYB10-1C
MYB10-1D
Supplementary Figure 4. Continued.
