# Supplementary material for: Whitening fruit by CRISPR/Cas9-mediated homoeolog-specific gene editing of MYB10-1B in strawberry (F. × ananassa)
Source: Hortic Res. 2025 Oct 15;13(1):uhaf272. doi: 10.1093/hr/uhaf272 (PMC12863208; doi:10.1093/hr/uhaf272)
Supplement: Web_Material_uhaf272 [file web_material_uhaf272.zip › Supplementary Figure 6.pptx]

## Slide 1
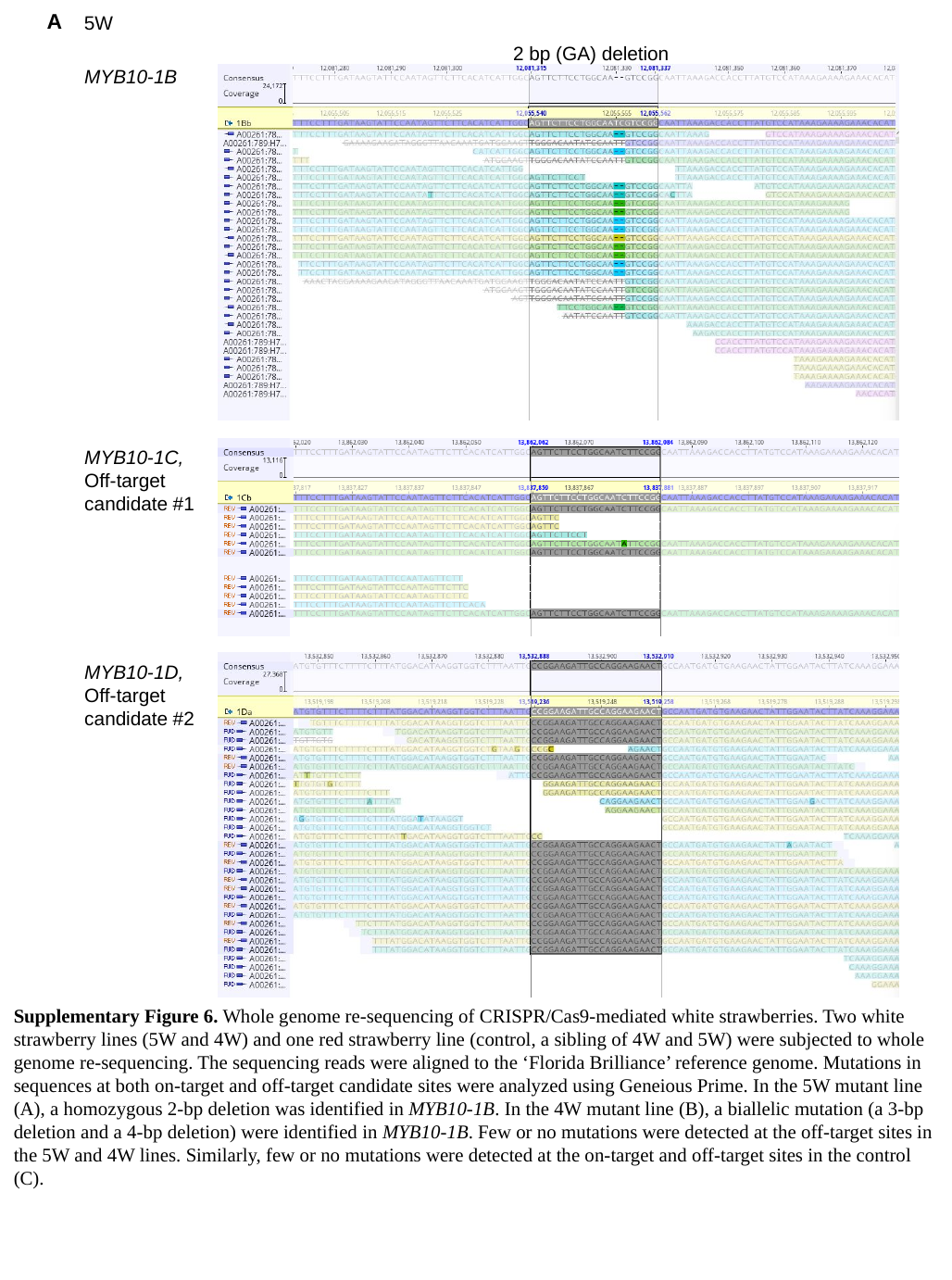

A
5W
2 bp (GA) deletion
MYB10-1B
MYB10-1C,
Off-target
candidate #1
MYB10-1D,
Off-target
candidate #2
Supplementary Figure 6. Whole genome re-sequencing of CRISPR/Cas9-mediated white strawberries. Two white strawberry lines (5W and 4W) and one red strawberry line (control, a sibling of 4W and 5W) were subjected to whole genome re-sequencing. The sequencing reads were aligned to the ‘Florida Brilliance’ reference genome. Mutations in sequences at both on-target and off-target candidate sites were analyzed using Geneious Prime. In the 5W mutant line (A), a homozygous 2-bp deletion was identified in MYB10-1B. In the 4W mutant line (B), a biallelic mutation (a 3-bp deletion and a 4-bp deletion) were identified in MYB10-1B. Few or no mutations were detected at the off-target sites in the 5W and 4W lines. Similarly, few or no mutations were detected at the on-target and off-target sites in the control (C).

## Slide 2
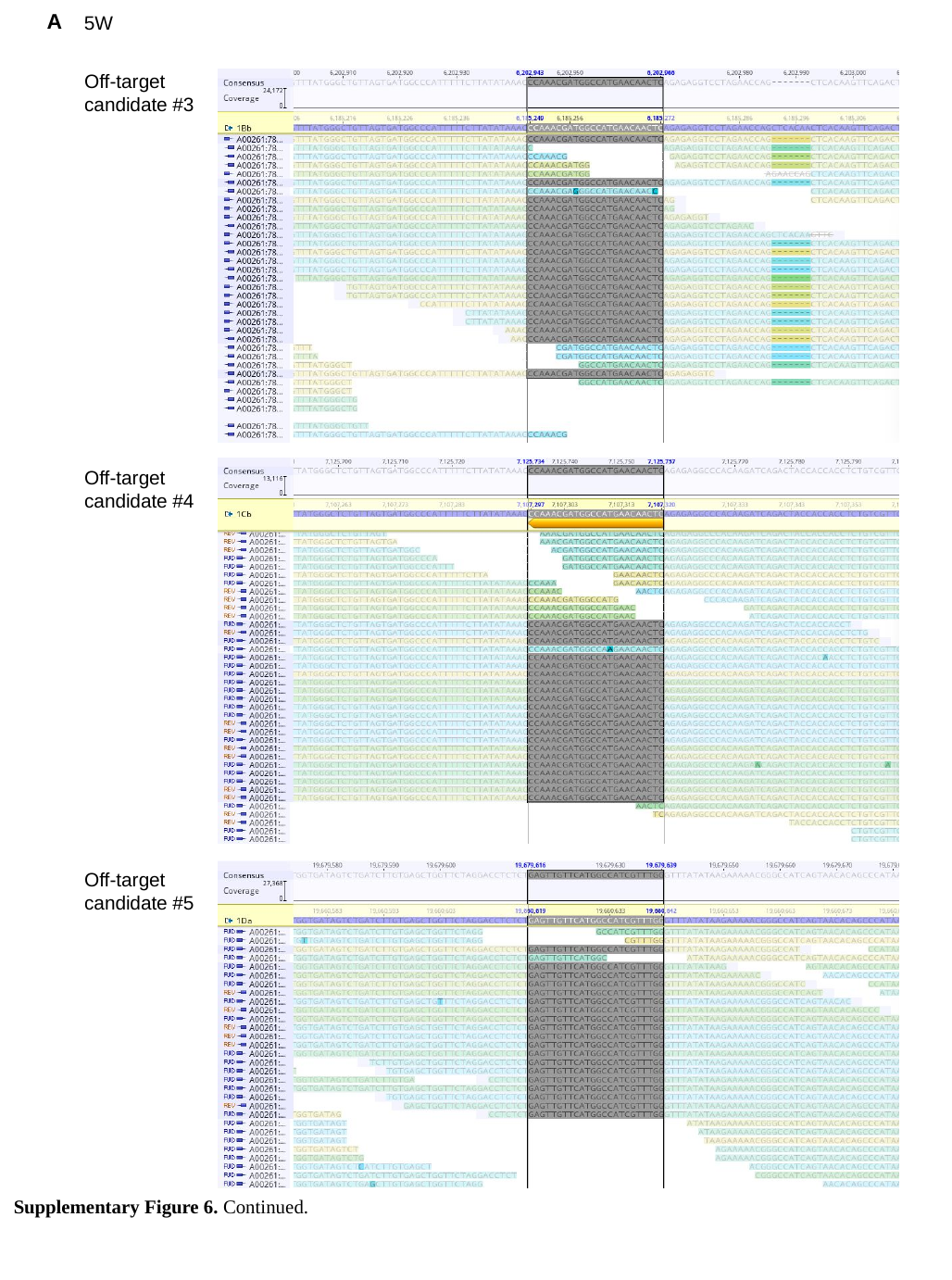

A
5W
Off-target
candidate #3
Off-target
candidate #4
Off-target
candidate #5
Supplementary Figure 6. Continued.

## Slide 3
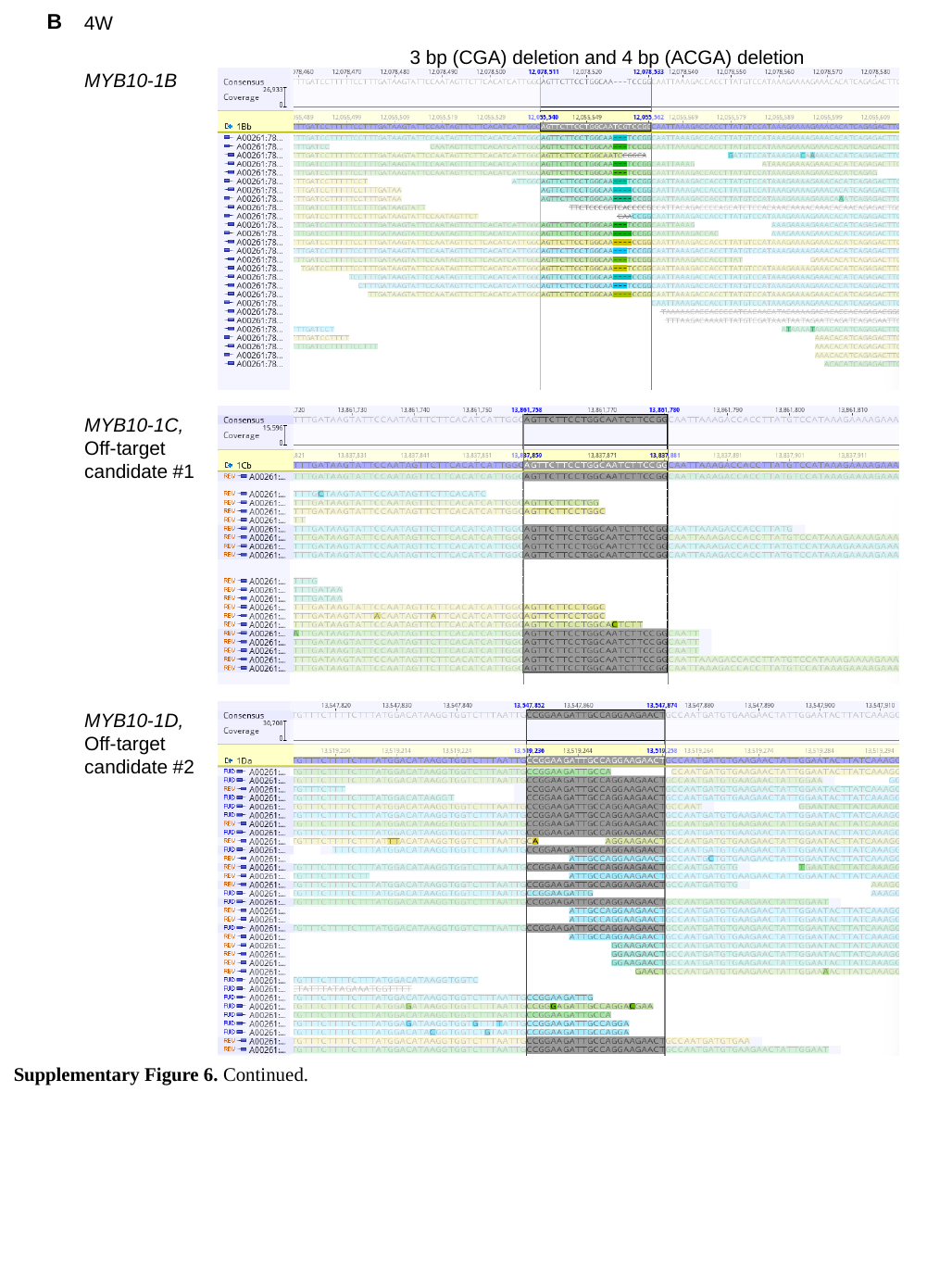

B
4W
3 bp (CGA) deletion and 4 bp (ACGA) deletion
MYB10-1B
2 bp (GA) deletion
MYB10-1C,
Off-target
candidate #1
MYB10-1D,
Off-target
candidate #2
Supplementary Figure 6. Continued.

## Slide 4
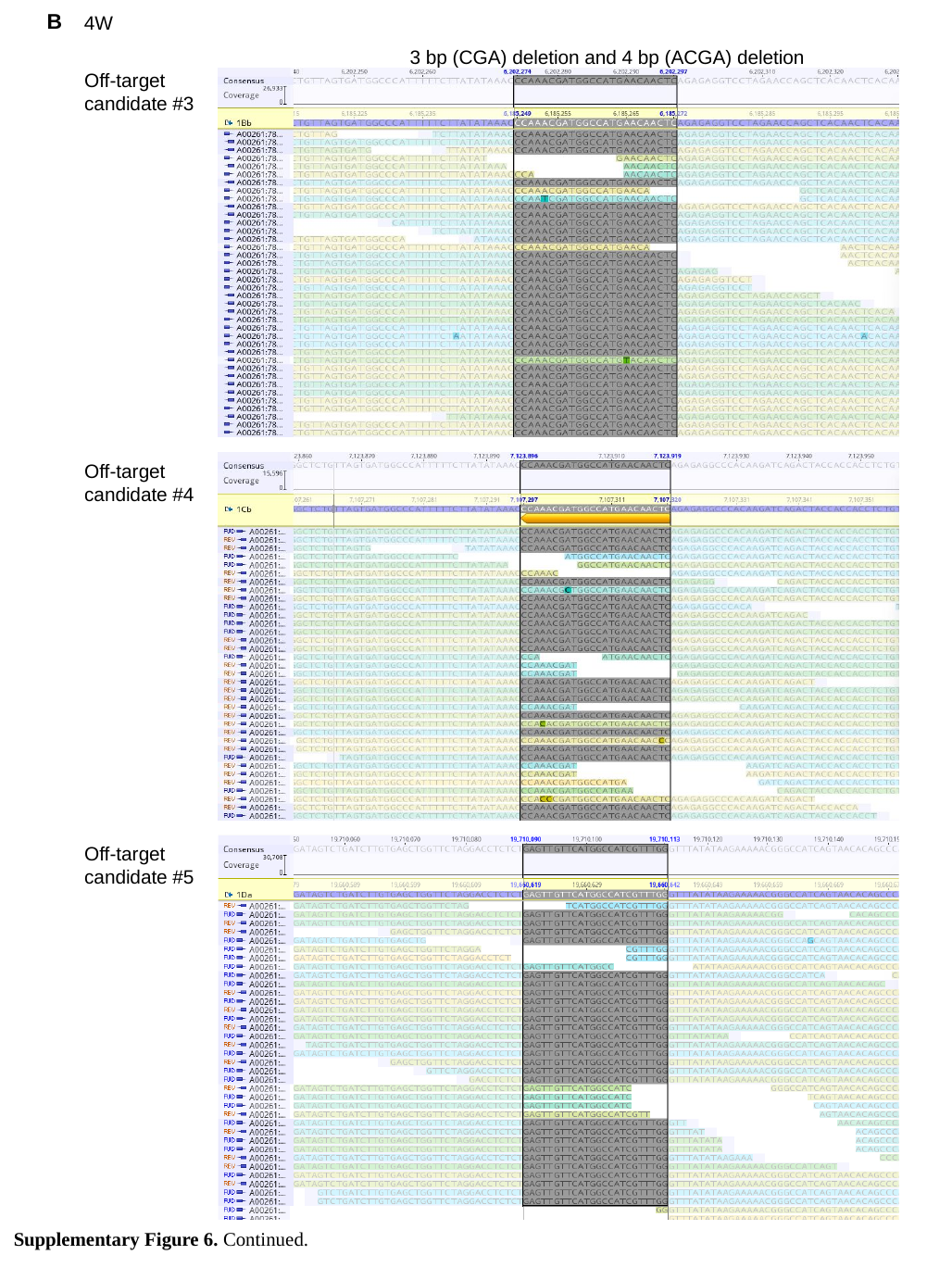

B
4W
3 bp (CGA) deletion and 4 bp (ACGA) deletion
Off-target
candidate #3
2 bp (GA) deletion
Off-target
candidate #4
Off-target
candidate #5
Supplementary Figure 6. Continued.

## Slide 5
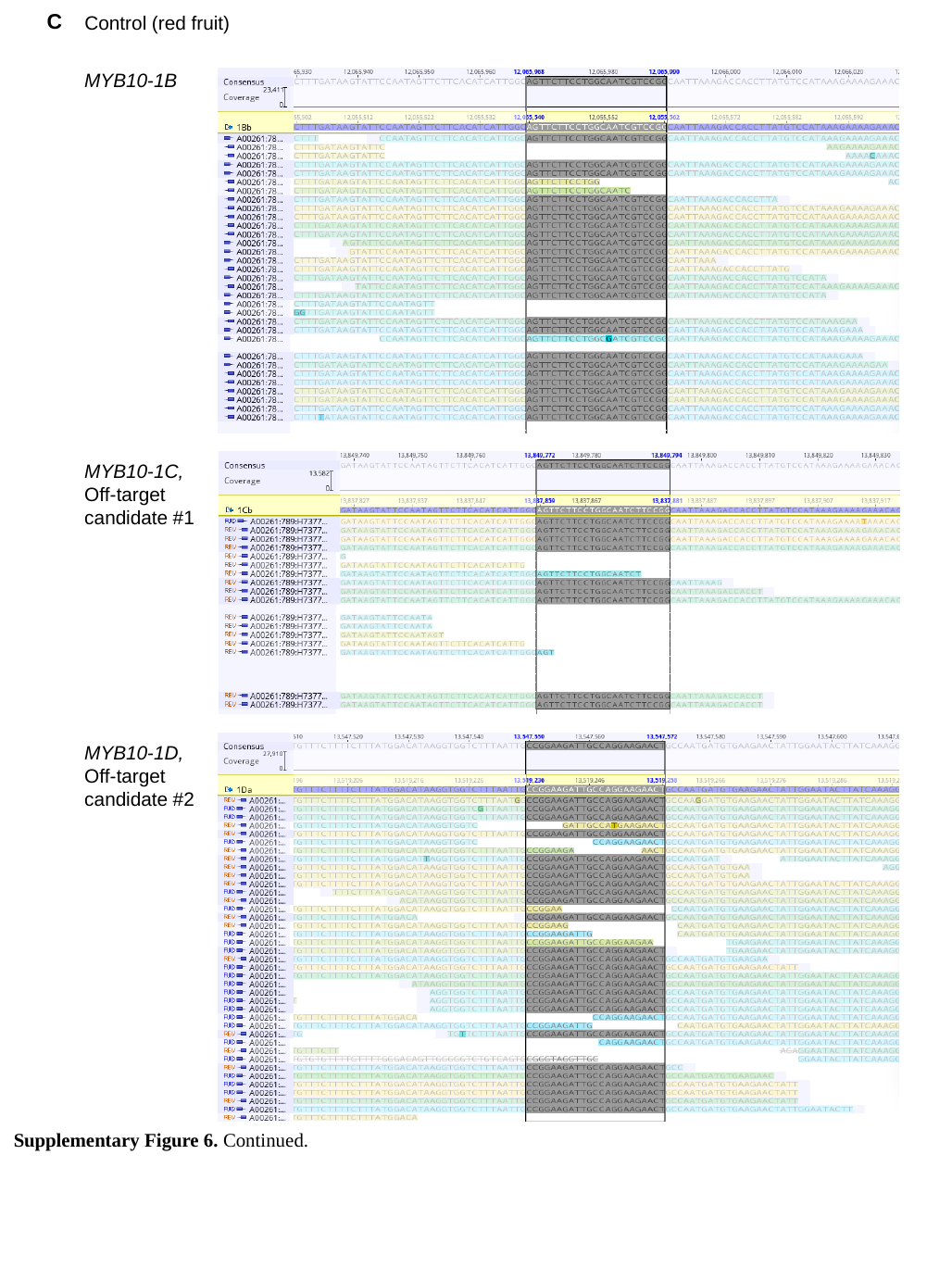

C
Control (red fruit)
MYB10-1B
2 bp (GA) deletion
MYB10-1C,
Off-target
candidate #1
MYB10-1D,
Off-target
candidate #2
Supplementary Figure 6. Continued.

## Slide 6
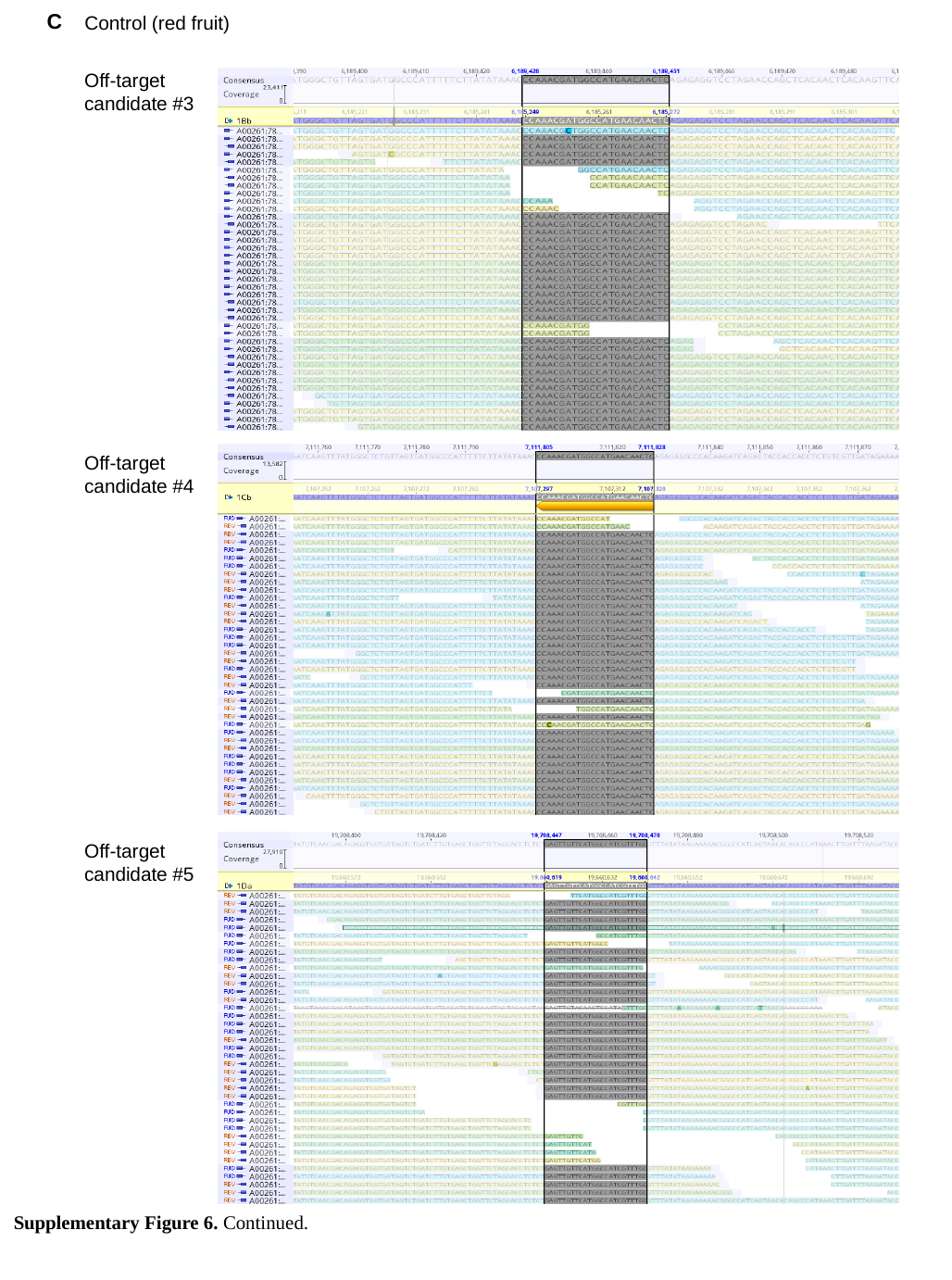

C
Control (red fruit)
Off-target
candidate #3
2 bp (GA) deletion
Off-target
candidate #4
Off-target
candidate #5
Supplementary Figure 6. Continued.
