# Supplementary material for: Whitening fruit by CRISPR/Cas9-mediated homoeolog-specific gene editing of MYB10-1B in strawberry (F. × ananassa)
Source: Hortic Res. 2025 Oct 15;13(1):uhaf272. doi: 10.1093/hr/uhaf272 (PMC12863208; doi:10.1093/hr/uhaf272)
Supplement: Web_Material_uhaf272 [file web_material_uhaf272.zip › Supplementary Figure 7.pptx]

## Slide 1
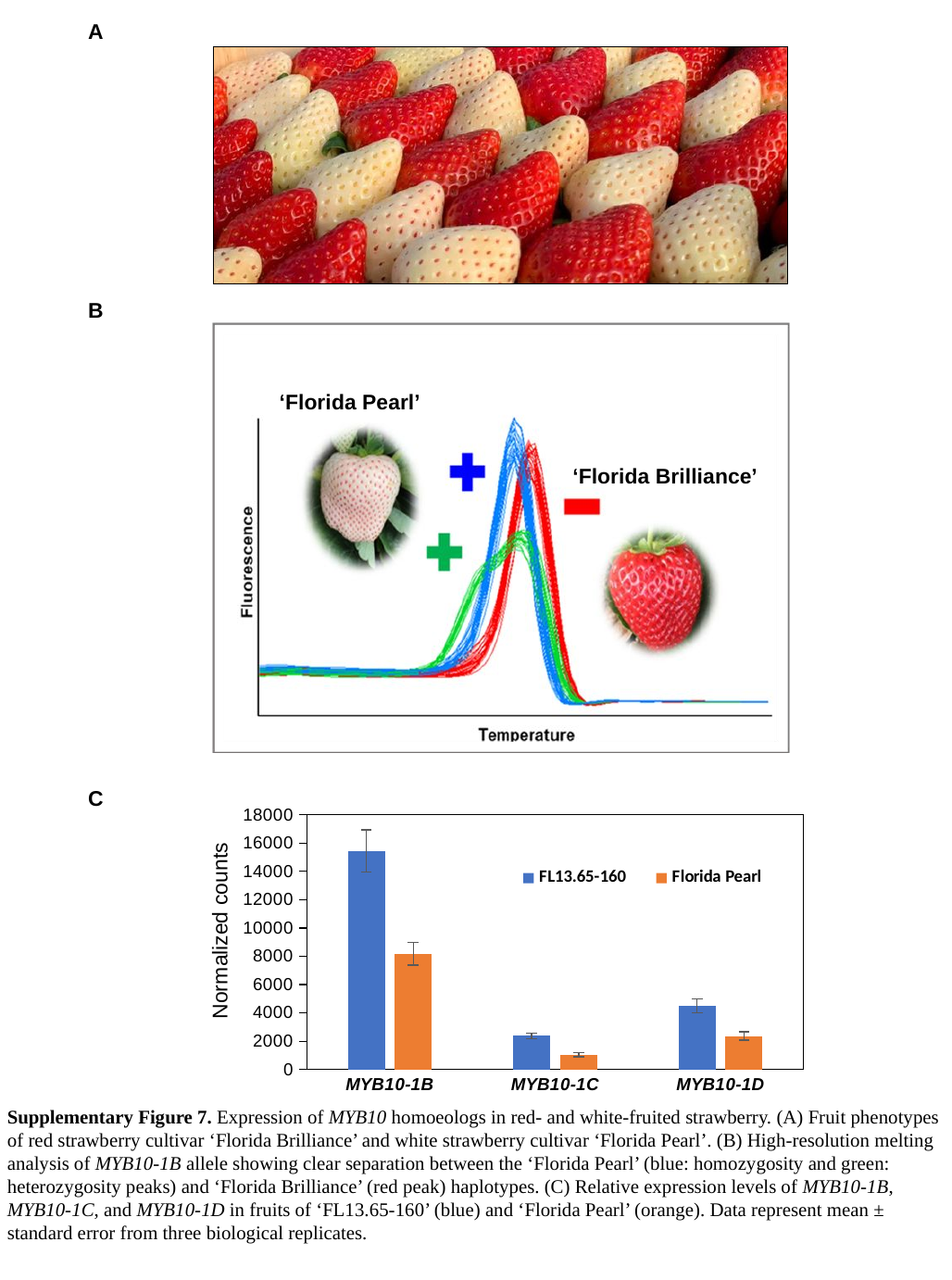

A
B
‘Florida Pearl’
‘Florida Brilliance’
C
### Chart
| Category | FL13.65-160 | Florida Pearl |
|---|---|---|
| MYB10-1B | 15452.0 | 8183.0 |
| MYB10-1C | 2368.0 | 1047.0 |
| MYB10-1D | 4488.0 | 2355.0 |Normalized counts
Supplementary Figure 7. Expression of MYB10 homoeologs in red- and white-fruited strawberry. (A) Fruit phenotypes of red strawberry cultivar ‘Florida Brilliance’ and white strawberry cultivar ‘Florida Pearl’. (B) High-resolution melting analysis of MYB10-1B allele showing clear separation between the ‘Florida Pearl’ (blue: homozygosity and green: heterozygosity peaks) and ‘Florida Brilliance’ (red peak) haplotypes. (C) Relative expression levels of MYB10-1B, MYB10-1C, and MYB10-1D in fruits of ‘FL13.65-160’ (blue) and ‘Florida Pearl’ (orange). Data represent mean ± standard error from three biological replicates.
