# Supplementary material for: Whitening fruit by CRISPR/Cas9-mediated homoeolog-specific gene editing of MYB10-1B in strawberry (F. × ananassa)
Source: Hortic Res. 2025 Oct 15;13(1):uhaf272. doi: 10.1093/hr/uhaf272 (PMC12863208; doi:10.1093/hr/uhaf272)
Supplement: Web_Material_uhaf272 [file web_material_uhaf272.zip › Supplementary Table 1.docx]

**Supplementary Table 1.** Transformation and gene editing efficiency in *Agrobacterium*-mediated transformation of strawberry (*F*. ×*ananassa*).

| **Repeat** | **Number of explants used for transformation** | **Number of shoots** | **Number of transgenic plants** | **Transformation efficiency (%)^z^** | **Number of edited plants** | **Mutation efficiency (%)^y^** |
| --- | --- | --- | --- | --- | --- | --- |
| 1 | 35 | 21 | 21 | 60.00 | 4 | 19.05 |
| 2 | 52 | 52 | 33 | 63.46 | 12 | 36.36 |

^z^ The transformation efficiency was calculated by dividing the number of transgenic plants with the number of explants used for transformation.

^y^ The mutation efficiency was calculated by dividing the number of edited plants with the number of transgenic plants.
