# Supplementary material for: Whitening fruit by CRISPR/Cas9-mediated homoeolog-specific gene editing of MYB10-1B in strawberry (F. × ananassa)
Source: Hortic Res. 2025 Oct 15;13(1):uhaf272. doi: 10.1093/hr/uhaf272 (PMC12863208; doi:10.1093/hr/uhaf272)
Supplement: Web_Material_uhaf272 [file web_material_uhaf272.zip › Supplementary Table 2.docx]

**Supplementary Table 2.** Chimeric mutants confirmation using HRM analysis.

|  | Tissues containing mutations | | | | | |
| --- | --- | --- | --- | --- | --- | --- |
|  | Leaf 1 | Leaf 2 | Leaf 3 | Leaf 4 | Sepal 1 | Sepal 2 |
| #6-C1-1 | O | X | X | X | X | O |
| #6-C1-2 | O | X | X | X | X | X |
| #8-C2-1 | O | X | X | X |  |  |
| #8-C2-2 | X | O | O | O |  |  |
| #8-C2-3 | X | X | O | O |  |  |
| #8-C2-4 | O | O | O | X |  |  |

Note: HRM analysis results were marked with an O for tissues showing peaks different from the wild-types, and an X for those that did not.
