# Supplementary material for: Whitening fruit by CRISPR/Cas9-mediated homoeolog-specific gene editing of MYB10-1B in strawberry (F. × ananassa)
Source: Hortic Res. 2025 Oct 15;13(1):uhaf272. doi: 10.1093/hr/uhaf272 (PMC12863208; doi:10.1093/hr/uhaf272)
Supplement: Web_Material_uhaf272 [file web_material_uhaf272.zip › Supplementary Table 3.docx]

**Supplementary Table 3.** Off-target candidates of the guide sequence for *MYB10-1B*.

|  | Sequence | Chromo-some | Position | Orientation |  |
| --- | --- | --- | --- | --- | --- |
| #1 | cAGTTCTTCCTGGCAATCtTCCGG | 1C | 13,837,857 | + | *MYB10-1C* |
| #2 | cAGTTCTTCCTGGCAATCtTCCGG | 1D | 13,519,235 | - | *MYB10-1D* |
| #3 | GAGTTgTTCaTGGCcATCGTtTGG | 1B | 6,185,248 | - |  |
| #4 | GAGTTgTTCaTGGCcATCGTtTGG | 1C | 7,107,296 | - |  |
| #5 | GAGTTgTTCaTGGCcATCGTtTGG | 1D | 19,660,618 | + |  |
